# Supplementary material for: TEAD2 initiates ground-state pluripotency by mediating chromatin looping
Source: EMBO J. 2024 Apr 11;43(10):3. doi: 10.1038/s44318-024-00086-5 (PMC11099042; doi:10.1038/s44318-024-00086-5)
Supplement: Supplementary file 1 — Appendix [file 44318_2024_86_MOESM1_ESM.pdf]

Appendix

TEAD2 initiates ground-state pluripotency by mediating chromatin looping

Table of contents:

Appendix Figure S1. TEAD2 has no effect on the core pluripotency establishment of ESCs. 2

Appendix Figure S2. Knockout of *Tead2* leads to an abnormal phenotype that can be maintained for an extended period during SL-to-2iL transition. 4

Appendix Figure S3. *Tead2* overexpression endows SL-ESCs with the expression of partial 2i-specific genes. 6

Appendix Figure S4. Analysis of BL-Hi-C data. 8

Appendix Figure S5. Supplementary data of ChIP-seq experiments. 10

Appendix Figure S6. Supplementary data of the QHR-4C experiments. 12

Appendix Figure S7. TEAD4 and TEAD2 are not redundant during SL-to-2iL transition. 14

Appendix Figure S8. Supplementary data for the DISCUSSION section. 16

Appendix Figure S1

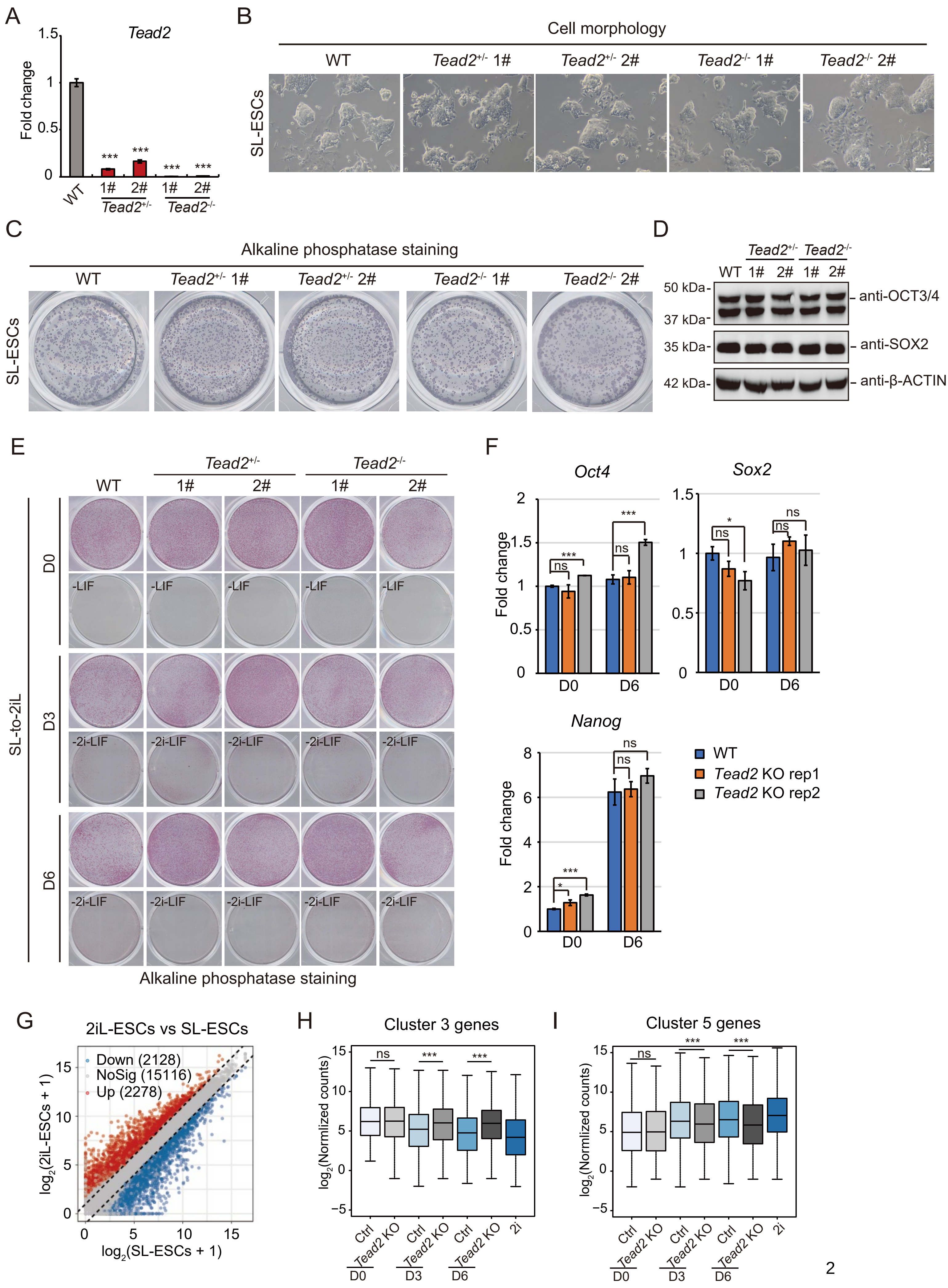

Appendix Figure S1. TEAD2 has no effect on the core pluripotency establishment of ESCs.

A. RT-qPCR testing the expression of *Tead2* in *Tead2*$^{-/-}$ and *Tead2*$^{+/-}$ SL-ESCs clones. Data are presented as the mean ± SD. *P*-values were determined by two-sided Student's *t*-test (***$p < 0.001$). n = 3 biological replicates.

B. Cellular morphology analysis of 1×10^5 *Tead2*$^{-/-}$ and *Tead2*$^{+/-}$ SL-ESCs grown on a 6-well plate coated with gelatin for three days. Scale bar, 100 µm.

C. AP-stained wells of *Tead2*$^{-/-}$ and *Tead2*$^{+/-}$ SL-ESCs after 5 days of culture.

D. Western blot analysis of the OCT4 and SOX2 protein levels in wild-type, *Tead2*$^{-/-}$, and *Tead2*$^{+/-}$ SL-ESCs.

E. Representative images of AP staining of wild-type and *Tead2*-knockout cells that were adapted to 2i conditions for days 0, 3, and 6. Cells were then induced for 5 days of differentiation in medium in the absence of LIF or 2i with LIF.

F. RT-qPCR testing the expression of pluripotent genes in wild-type and *Tead2*-knockout cells on day 0 and day 6 of the transition. Data are presented as the mean ± SD. Indicated significances are testing using Student's *t*-test analyses (*$p < 0.05$, ***$p < 0.001$). n = 3 biological replicates.

G. Volcano plots showing differential gene expression (fold change > 2; *q*-value < 0.05) between 2iL-ESCs and SL-ESCs.

H and I. Boxplots showing expression level of genes in cluster 3 (H) and cluster 5 (I) between wild-type and *Tead2*-knockout cells at day 0, 3, and 6 of the transition and 2iL-ESCs. The centerline indicates the median value, while the box and whiskers represent the interquartile range (IQR) and 1.5 × IQR, respectively, n = 472 in (H), n = 1,210 in (I). ***$p < 0.001$. *P*-value was calculated by using Mann-Whitney U test.

Appendix Figure S2

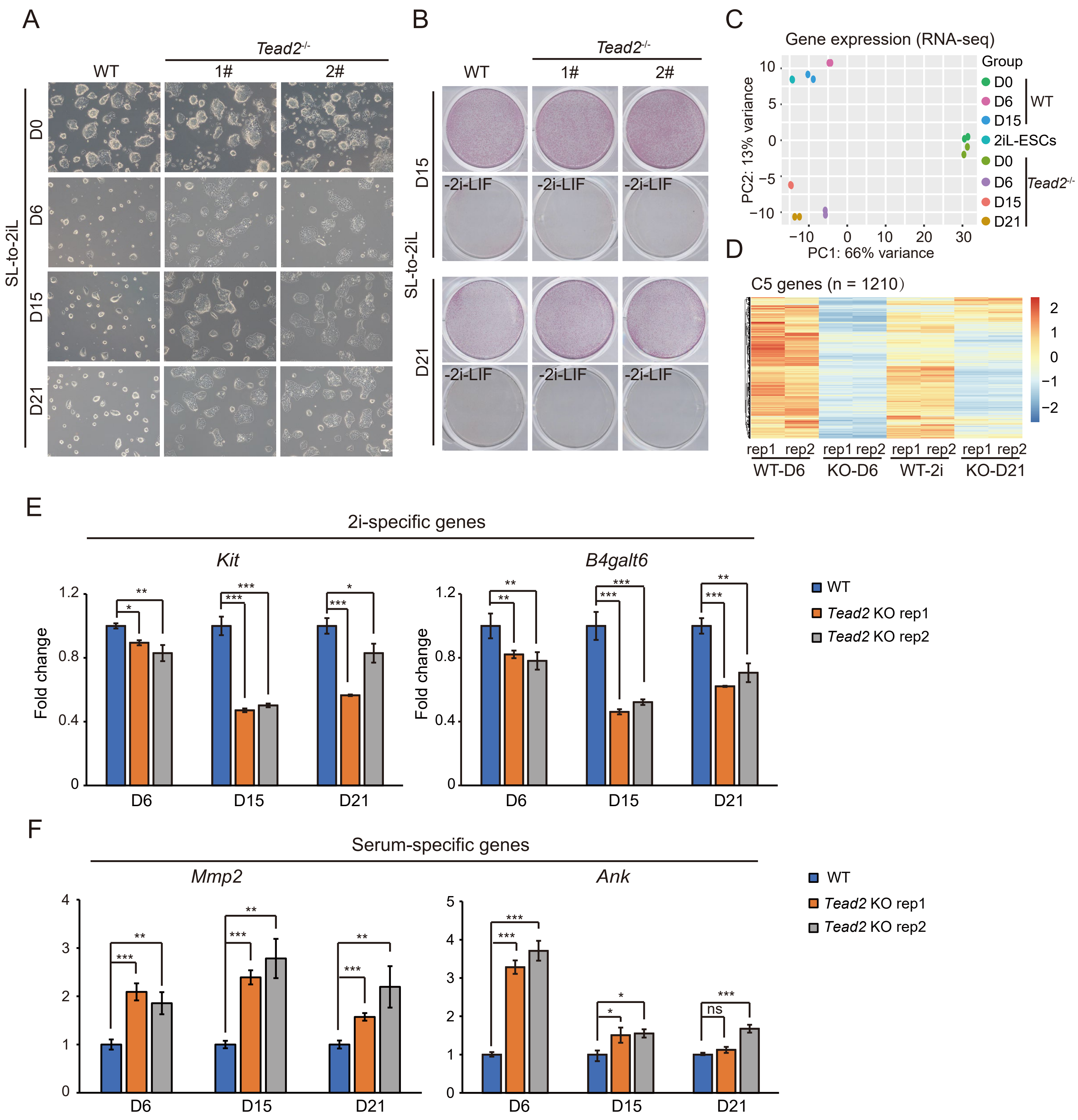

Appendix Figure S2. Knockout of *Tead2* leads to an abnormal phenotype that can be maintained for an extended period during SL-to-2iL transition.

A. Representative cellular morphology of wild-type ESCs and *Tead2*-knockout ESCs during the SL-to-2iL transition at days 0, 6, 15, and 21. Scale bar, 100 μm.

B. Representative images of AP staining of wild-type and *Tead2*-knockout cells that were adapted to 2i conditions for 15 and 21 days. Cells were then induced for differentiation for 5 days in medium in the absence of LIF or 2i with LIF.

C. PCA of the RNA-seq data from wild-type ESCs and *Tead2*-knockout ESCs collected at different time points during the SL-to-2iL transition.

D. Heatmap showing the expression of cluster 5 genes in wild-type and *Tead2*-knockout cells on day 6 and 21 during the transition.

E and F. RT-qPCR testing the expression of 2i-specific genes (E) and serum-specific genes (F) in wild-type and *Tead2*-knockout cells on days 6, 15, and 21 of the transition. Data are presented as the mean ± SD. Indicated significances are tested using Student's *t*-test analyses (*$p < 0.05$, **$p < 0.01$, ***$p < 0.001$). n = 3 biological replicates. The fold changes of these specific gene expressions after knocking *Tead2* out were calculated by using the wild-type of D6, D15, and D21 as controls, respectively.

Appendix Figure S3

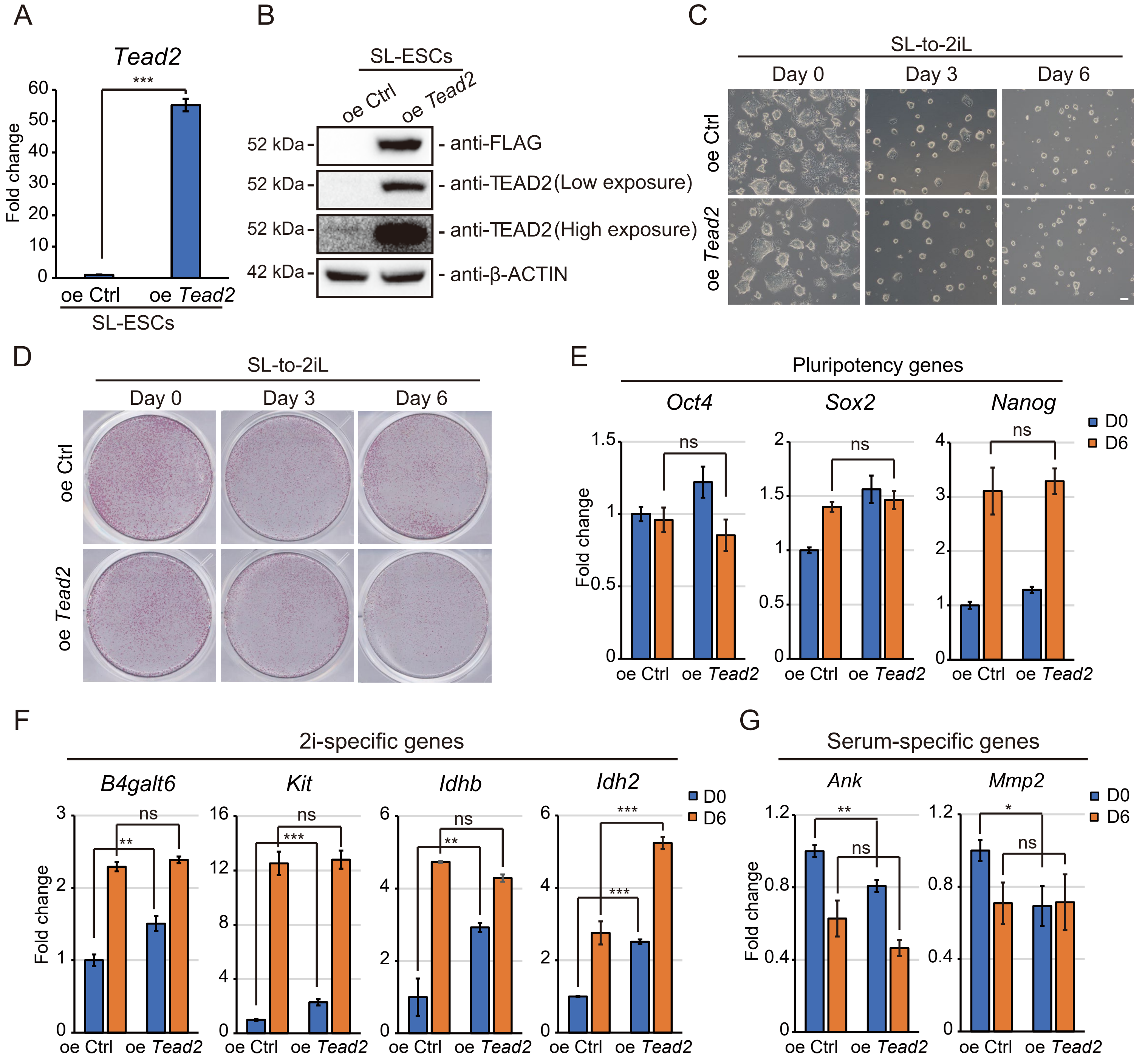

Appendix Figure S3. *Tead2* overexpression endows SL-ESCs with the expression of partial 2i-specific genes.

A and B. RT-qPCR (A) and Western blot (B) analysis examining *Tead2* overexpression in SL-ESCs. Data are presented as the mean ± SD. Indicated significances are tested using Student's *t*-test analyses (***$p < 0.001$). n = 3 biological replicates.

C. Representative cellular morphology of ESC colonies with the Flag control and Flag-*Tead2* overexpression during the SL-to-2iL transition at days 0, 3, and 6, respectively. Scale bar, 100 μm.

D. Representative images of AP staining of the cells with the Flag control and Flag-*Tead2* overexpression that were adapted to 2i conditions for 0, 3, and 6 days.

E-G. RT-qPCR analysis testing the expression of pluripotency genes (E), 2i-specific genes (F), and serum-specific genes (G) in Flag control and Flag-*Tead2* overexpression cells on day 0 and day 6 of the transition. Data are presented as the mean ± SD. Indicated significances are tested using Student's *t*-test analysis (*$p < 0.05$, **$p < 0.01$, ***$p < 0.001$). n = 3 biological replicates.

Appendix Figure S4

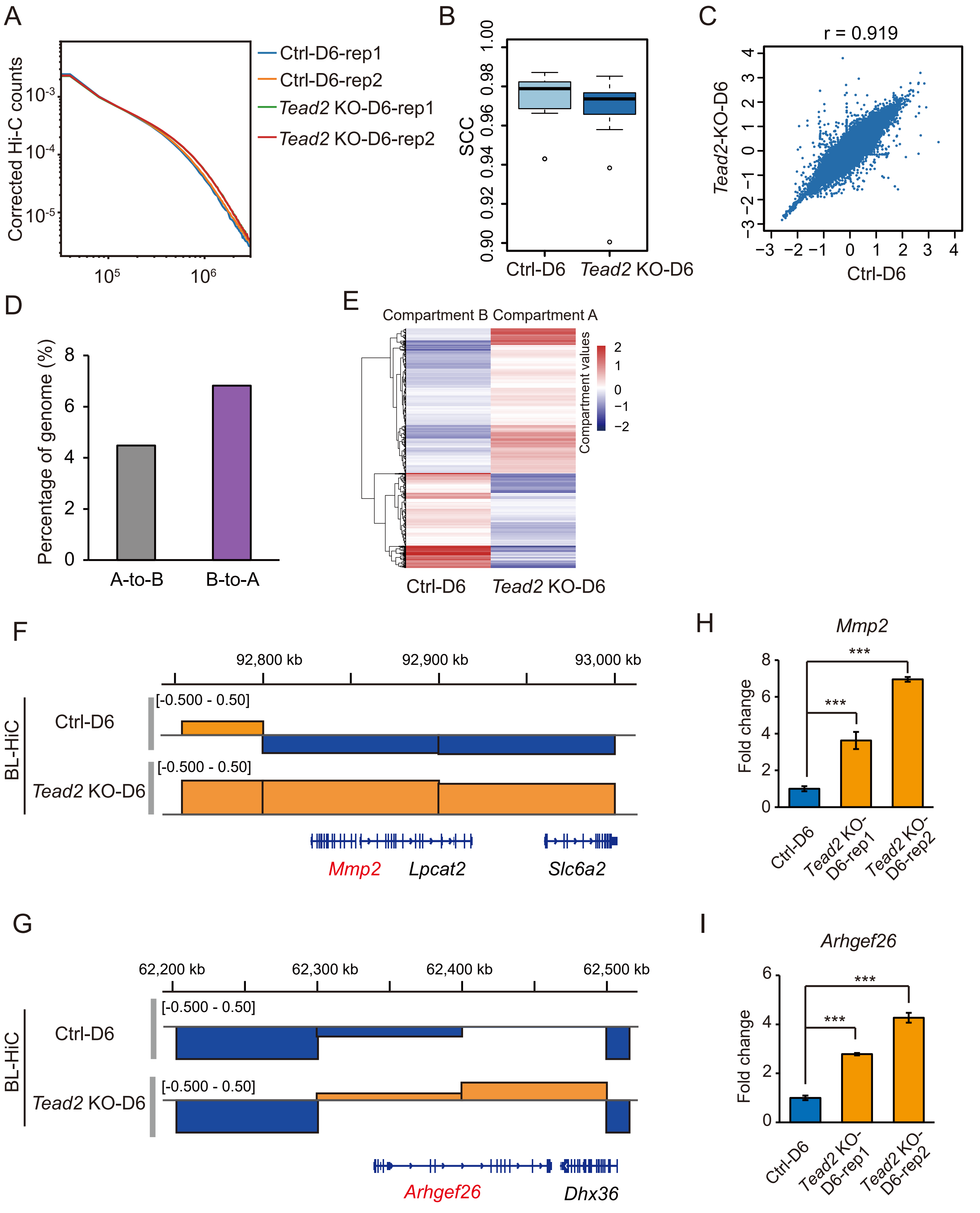

Appendix Figure S4. Analysis of BL-Hi-C data.

A. Contact frequency distance curves obtained from BL-Hi-C data from both wild-type and *Tead2*-knockout cells at day 6 of the SL-to-2iL transition (n = 2 biological replicates).

B. Boxplots showing reproducibility measured as SCC scores for all pairs of replicates. The centerline indicates the median value, while the box and whiskers represent the interquartile range (IQR) and 1.5 × IQR, respectively. n = 22.

C. TAD analysis of wild-type and *Tead2*-knockout cells at day 6 of the SL-to-2iL transition.

D. Barplots showing the percentage of A/B compartment switches between wild-type and *Tead2*-knockout cells at day 6 of the SL-to-2iL transition.

E. Heatmaps showing the A/B compartment shifted regions between wild-type and *Tead2*-knockout cells at day 6 of the SL-to-2iL transition.

F and G. A/B compartment analysis at *Mmp2* (F) and *Arhgef26* (G) loci in both wild-type and *Tead2*-knockout cells at day 6 of the SL-to-2iL transition from BL-Hi-C experiments.

H and I. RT-qPCR detecting the expression levels of *Mmp2* (H) and *Arhgef26* (I) genes. Data are presented as the mean ± SD. *P*-values were determined by two-sided Student's *t*-test (****p* < 0.001). n = 3 biological replicates.

Appendix Figure S5

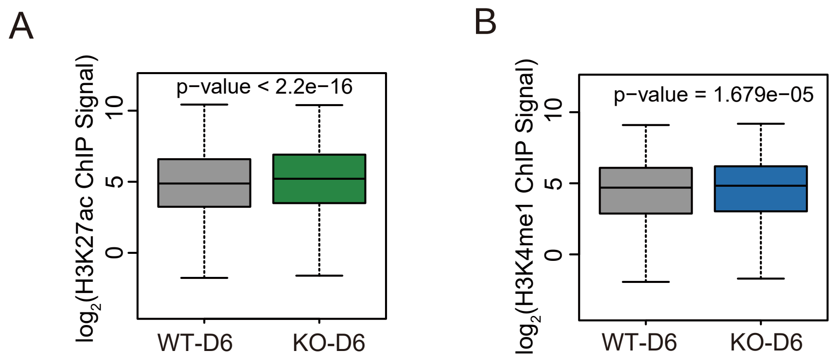

Appendix Figure S5. Supplementary data of ChIP-seq experiments.

A and B. Boxplots showing the CUT&Tag signal of both H3K27ac (A) and H3K4me1 (B) at TEAD2 binding sites in wild-type and *Tead2*-knockout cells, respectively, at day 6 of the SL-to-2iL transition. The centerline indicates the median value, while the box and whiskers represent the interquartile range (IQR) and 1.5 × IQR, respectively. n = 10,315. *P*-value was calculated by using Mann-Whitney U test.

Appendix Figure S6

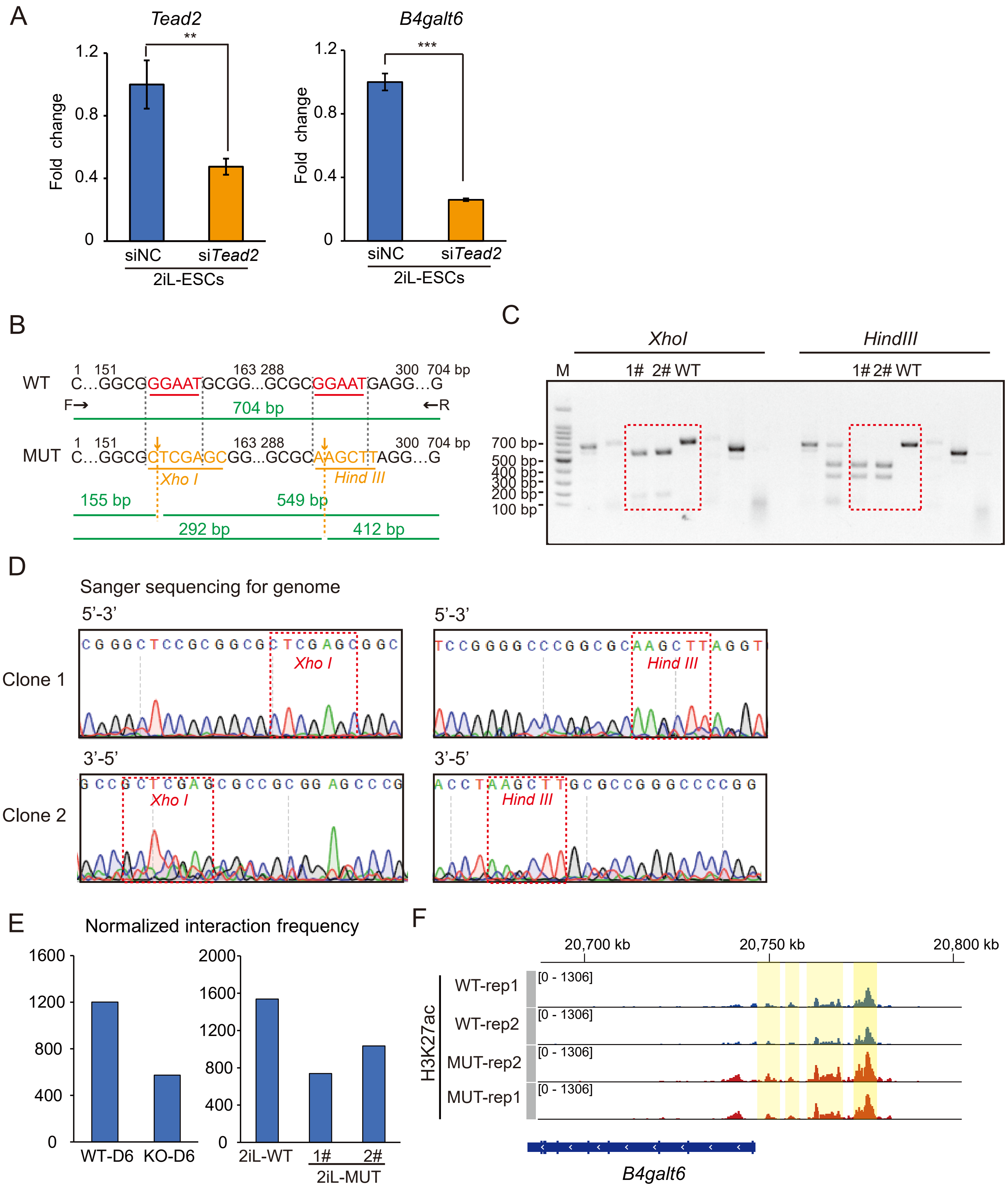

Appendix Figure S6. Supplementary data of the QHR-4C experiments.

A. RT-qPCR determining the expression of *Tead2* and *B4galt6* in 2iL-ESCs. Data are presented as the mean ± SD. Indicated significances are tested using Student's *t*-test analysis (**$p < 0.01$, ***$p < 0.001$). n = 3 biological replicates.

B. Restriction enzyme digestion strategy for identifying mutant clones.

C. Genomic PCR and enzyme digestion to verify corrected clones.

D. Sanger sequencing testing the region containing two TEAD2 motifs in wild-type and two homozygous mutant clones of 2iL-ESCs.

E. Barplot showing the normalized interaction frequency between the promoters and enhancers of *B4galt6* in wild-type and two mutant 2iL-ESCs, wild-type and *Tead2*-knockout cells at day 6 of the transition.

F. Genomic views of enrichment for H3K27ac at the *B4galt6* gene in wild-type and two homozygous mutant clones of 2iL-ESCs.

Appendix Figure S7

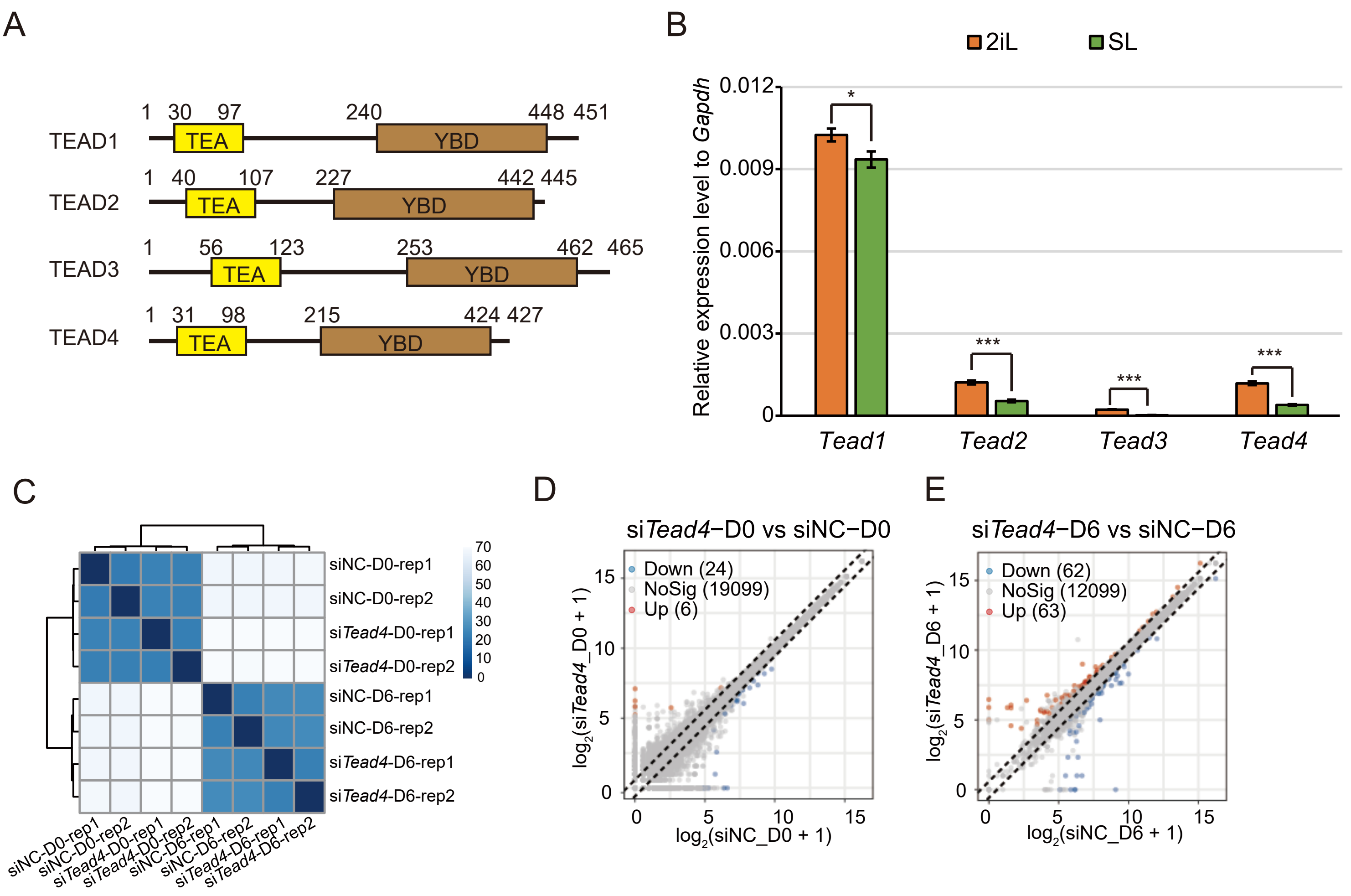

Appendix Figure S7. TEAD4 and TEAD2 are not redundant during SL-to-2iL transition.

A. Schematic of the overall structure of the mammalian TEAD factors. The four TEADs present an overall homology and are divided into a TEA domain at the N-terminus (67 aa) and a C-terminal YAP/TAZ binding domain (YBD) (about 215 aa). Both domains are linked by a sequence of about 117–143 amino acids which has a low homology across the four TEADs. TEA: TEA DNA binding domain. YBD: YAP binding domain.

B. RT-qPCR analysis testing the expression of *Tead1-4* in 2iL- and SL-ESCs. Data are presented as the mean ± SD. Indicated significances are tested using Student's *t*-test analyses (*$p < 0.05$, ***$p < 0.001$). n = 3 biological replicates.

C. Hierarchical cluster analysis (HCA) and heatmaps of control and *Tead4*-depleted cells at day 0 and day 6 of the transition. The heatmaps were based on rlog-transformed and DESeq2-normalized expression data. The color key shows the Euclidean distances between samples.

D and E. Volcano plots showing differential gene expression (fold change > 2; *q*-value < 0.05) between control and *Tead4*-depleted cells at day 0 (D) and day 6 (E) of the SL-to-2iL transition.

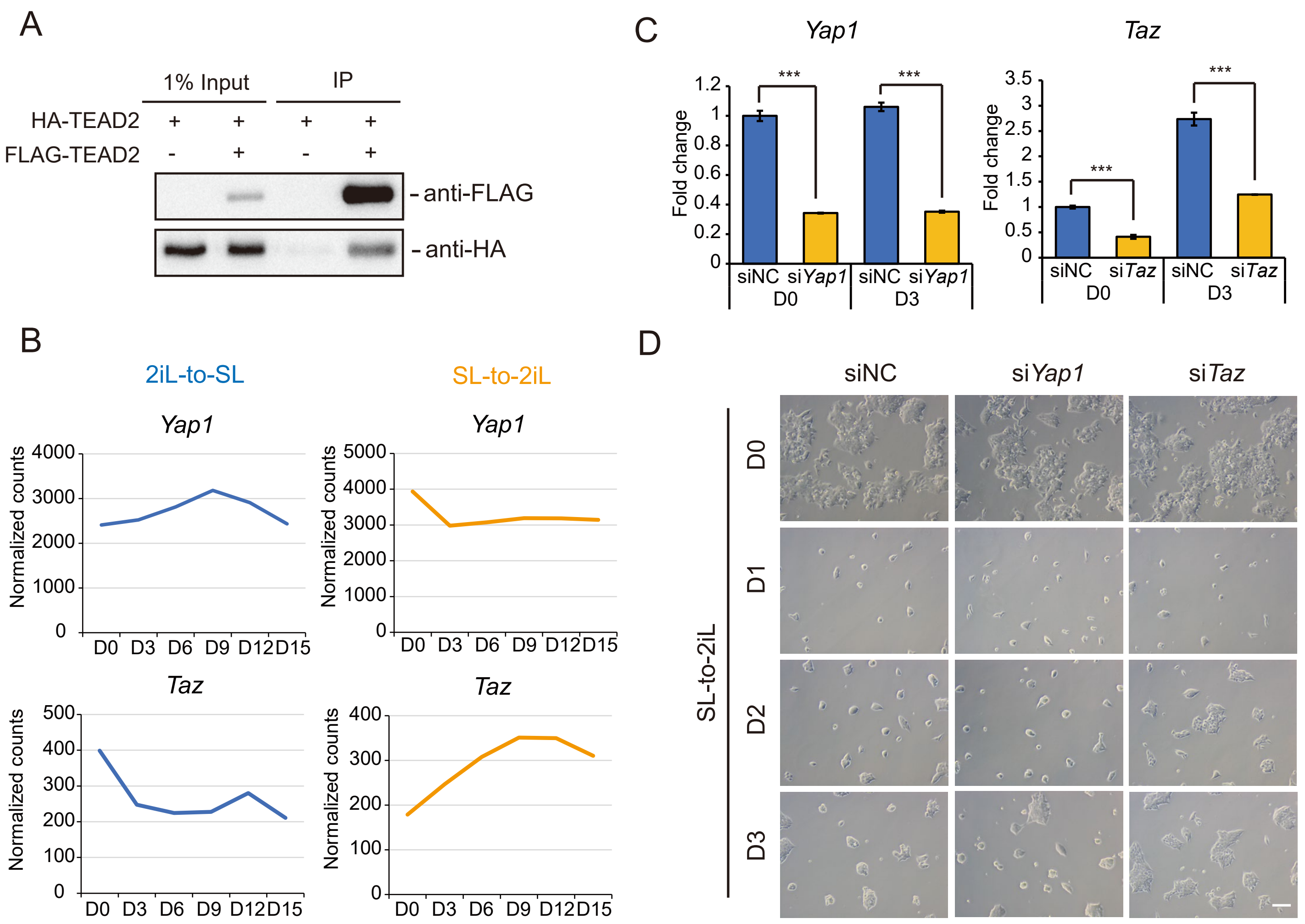
A
1% Input
IP
HA-TEAD2
FLAG-TEAD2
anti-FLAG
anti-HA
B
2iL-to-SL
SL-to-2iL
Yap1
Taz
Normalized counts
D0 D3 D6 D9 D12 D15
C
Yap1
Taz
Fold change
siNC
siYap1
siTaz
D0
D3
D
siNC
siYap1
siTaz
SL-to-2iL
D0
D1
D2
D3

Appendix Figure S8. Supplementary data for the DISCUSSION section.

A. Western blot showing FLAG-immunoprecipitation of FLAG-tagged TEAD2 and HA-tagged TEAD2 protein from the transfected cells.

B. Expression patterns for *Yap1* and *Taz* during the conversion between 2iL-ESCs and SL-ESCs.

C. RT-qPCR testing knockdown efficiencies for *Yap1* and *Taz* during the SL-to-2iL transition. Cells were treated with specific siRNAs every 3 days along with control siRNA. Data are presented as the mean ± SD. Indicated significances are tested using Student's *t*-test analyses (***$p < 0.001$). n = 3 biological replicates.

D. Representative images of cells transfected with siNC (negative control) and siRNAs targeting *Yap1* and *Taz*, respectively, during the SL-to-2iL process. Scale bar, 100 μm.
