# Supplementary figures and images for: TEAD2 initiates ground-state pluripotency by mediating chromatin looping
Source: EMBO J. 2024 Apr 11;43(10):3. doi: 10.1038/s44318-024-00086-5 (PMC11099042; doi:10.1038/s44318-024-00086-5)

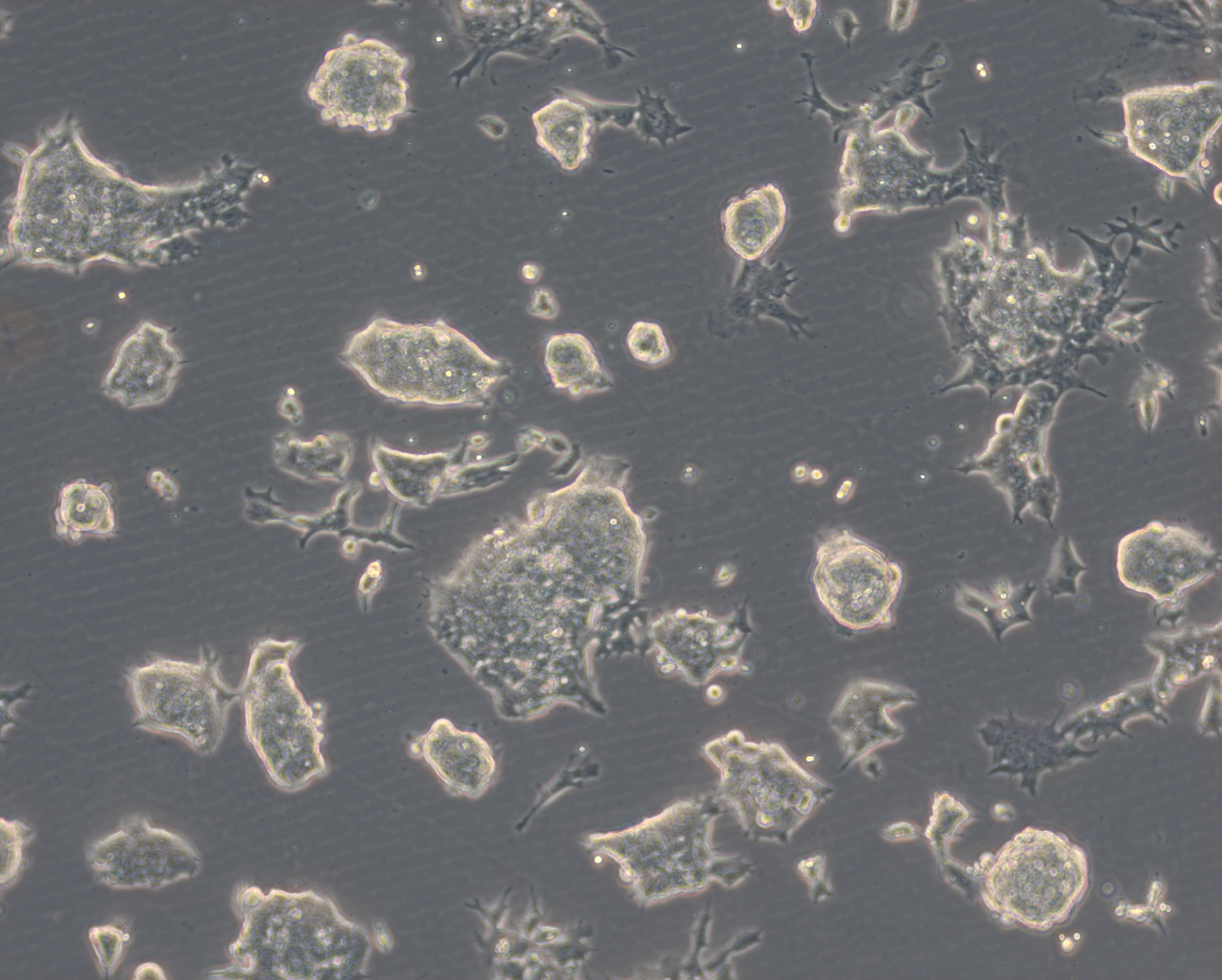

Supplement: Supplementary file 13 — Source data Fig. 2 [file 44318_2024_86_MOESM13_ESM.zip › Figure 2/Figure 2D/D0-siEsrrb.jpg]

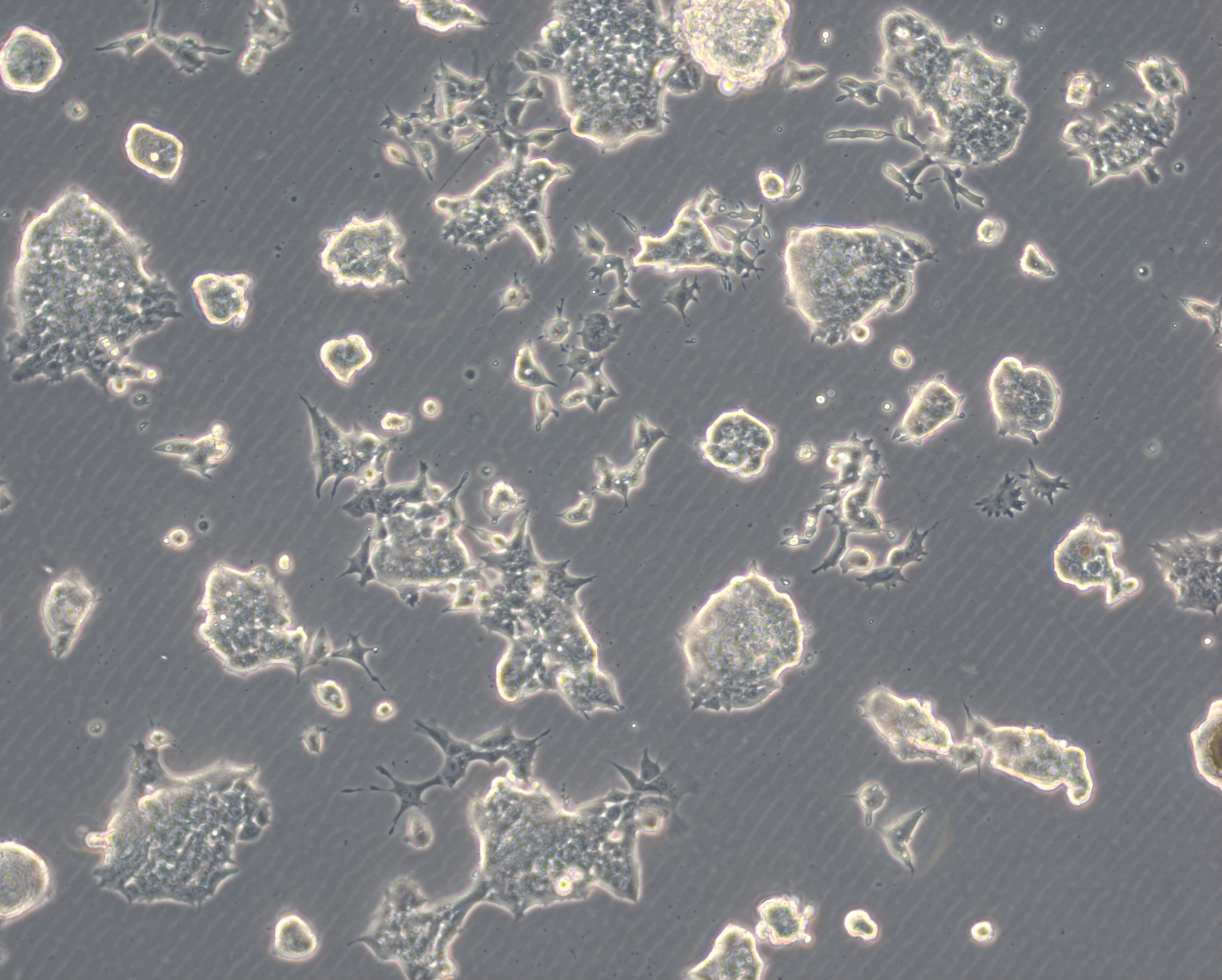

Supplement: Supplementary file 13 — Source data Fig. 2 [file 44318_2024_86_MOESM13_ESM.zip › Figure 2/Figure 2D/D0-siNC.jpg]

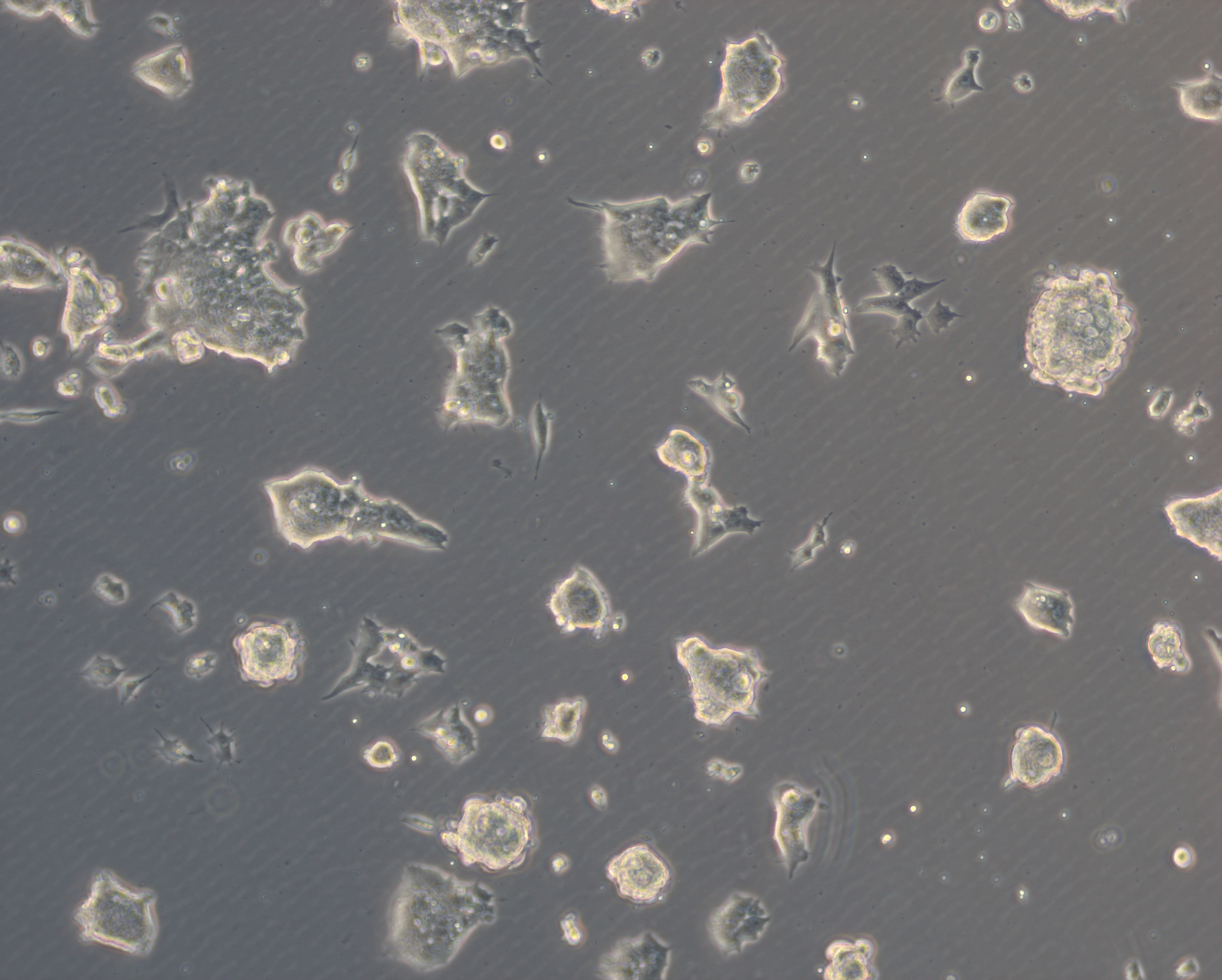

Supplement: Supplementary file 13 — Source data Fig. 2 [file 44318_2024_86_MOESM13_ESM.zip › Figure 2/Figure 2D/D0-siNr5a2.jpg]

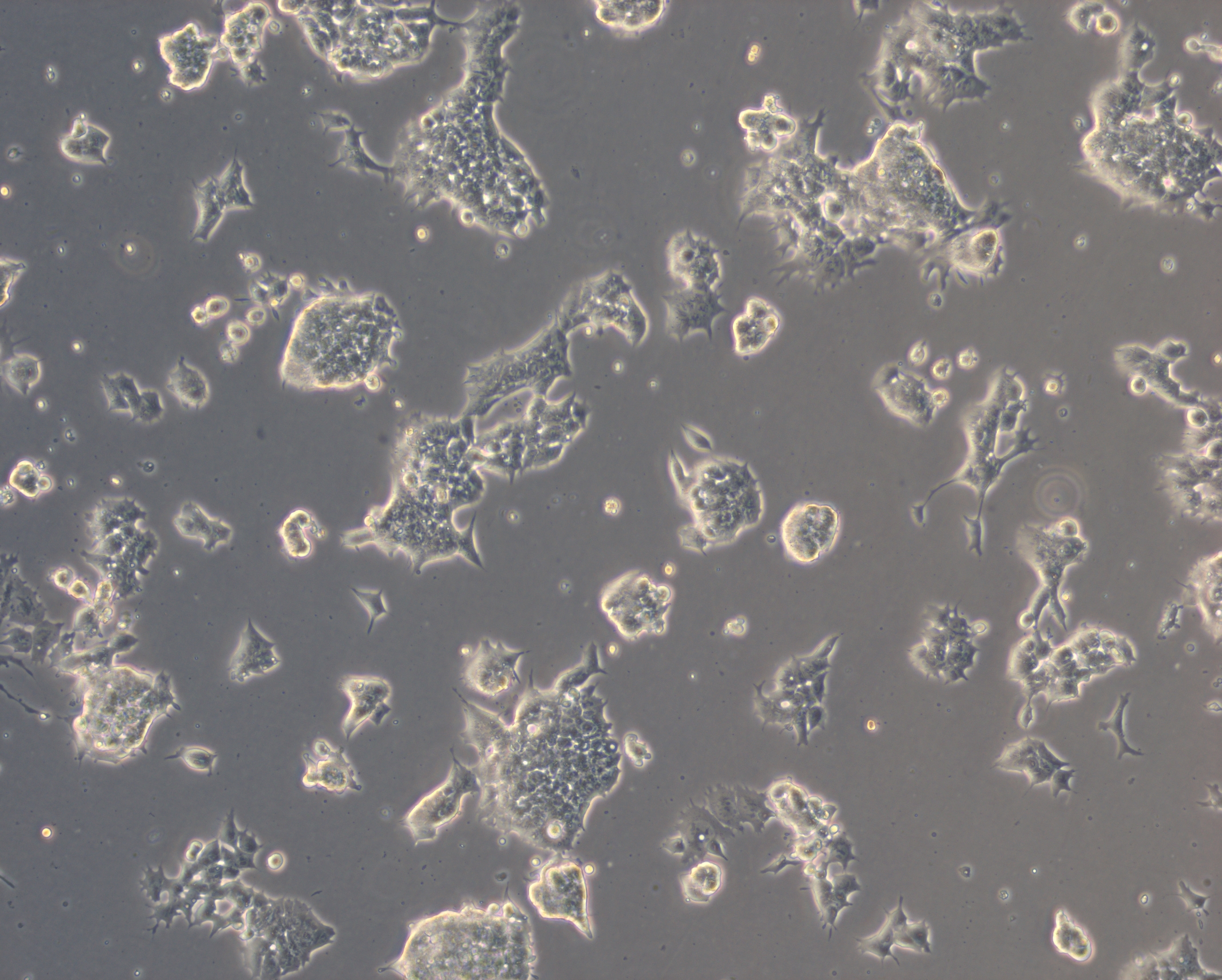

Supplement: Supplementary file 13 — Source data Fig. 2 [file 44318_2024_86_MOESM13_ESM.zip › Figure 2/Figure 2D/D0-siTead2.jpg]

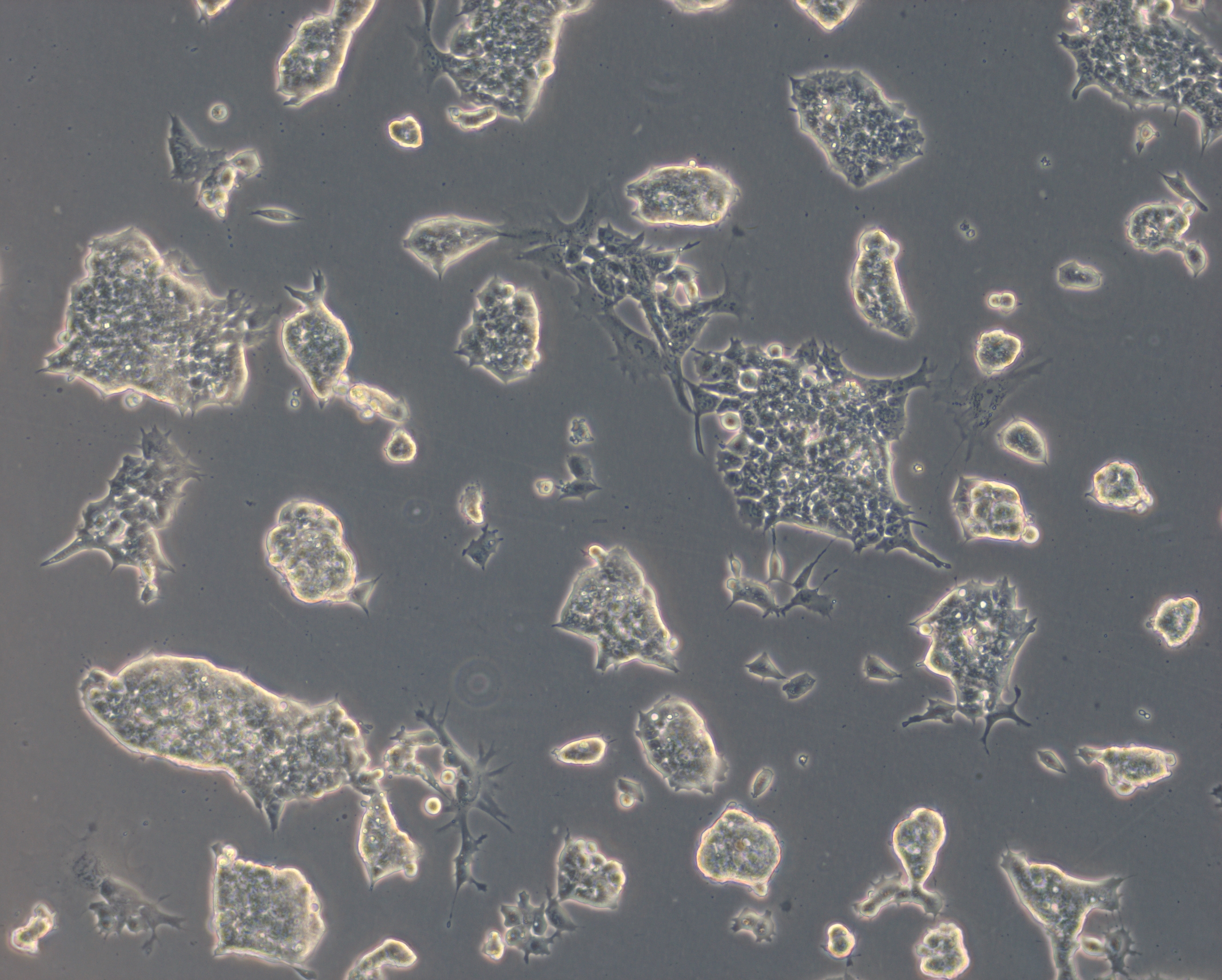

Supplement: Supplementary file 13 — Source data Fig. 2 [file 44318_2024_86_MOESM13_ESM.zip › Figure 2/Figure 2D/D0-siTead4.jpg]

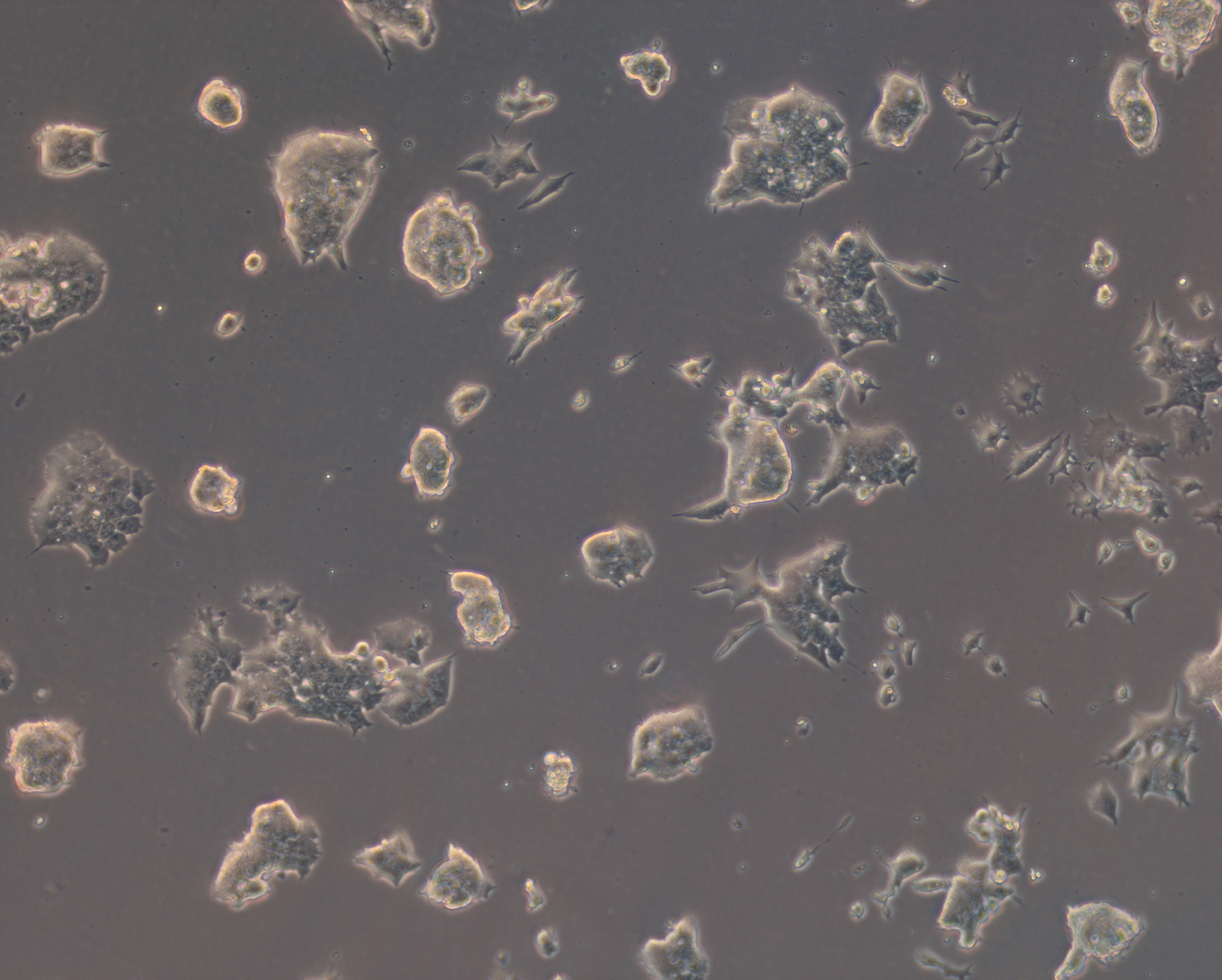

Supplement: Supplementary file 13 — Source data Fig. 2 [file 44318_2024_86_MOESM13_ESM.zip › Figure 2/Figure 2D/D0-siTfcp2l1.jpg]

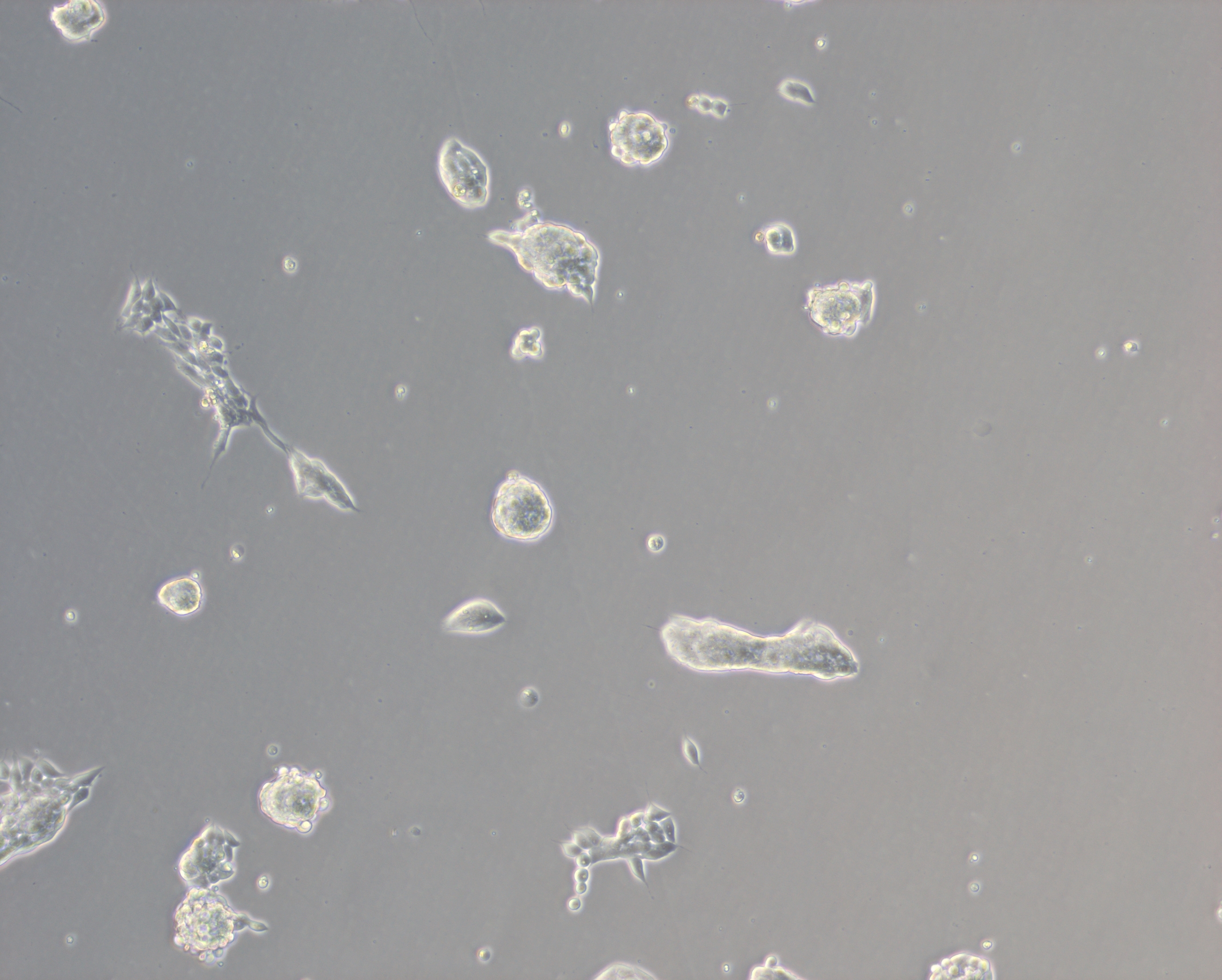

Supplement: Supplementary file 13 — Source data Fig. 2 [file 44318_2024_86_MOESM13_ESM.zip › Figure 2/Figure 2D/D3-siErssb.jpg]

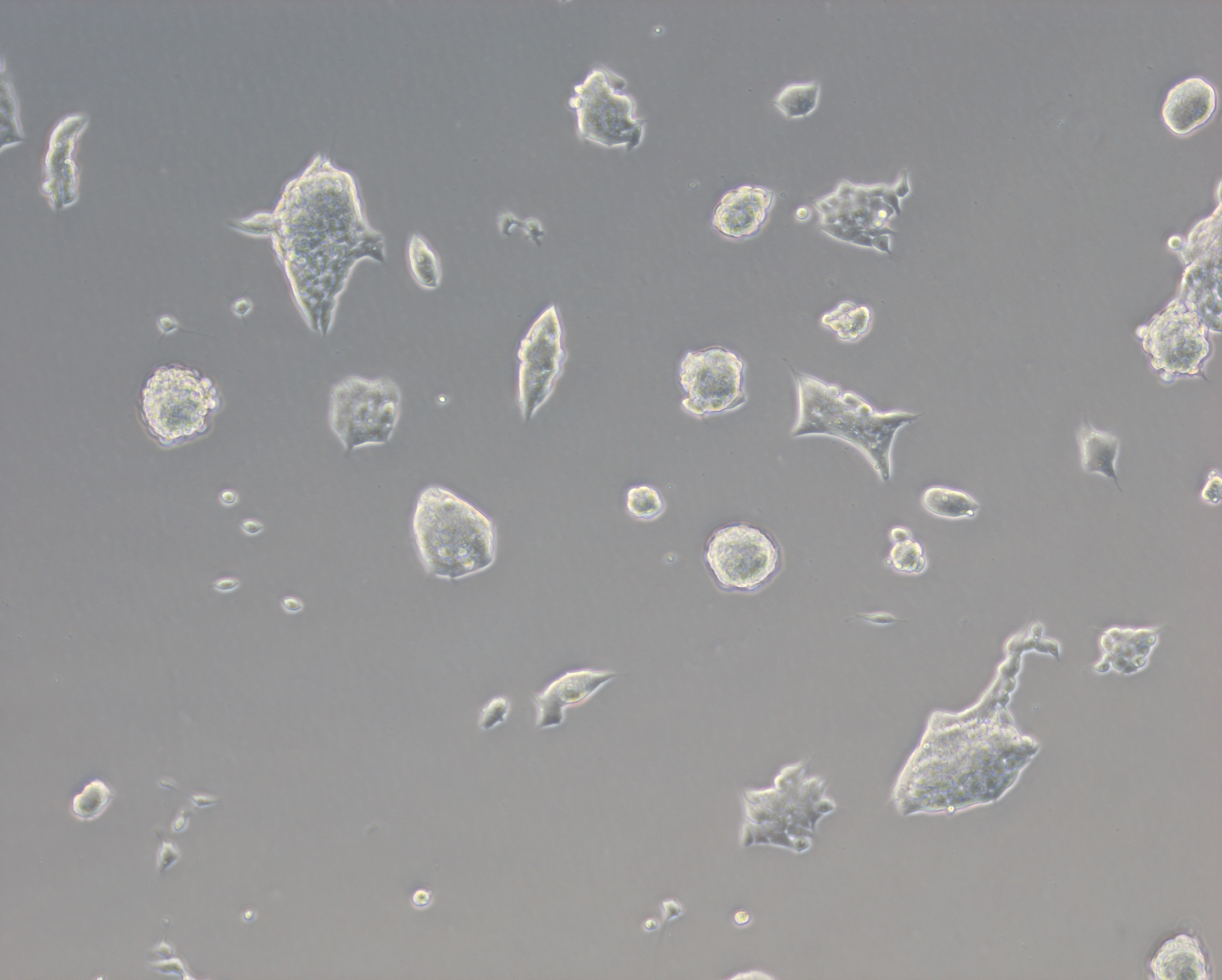

Supplement: Supplementary file 13 — Source data Fig. 2 [file 44318_2024_86_MOESM13_ESM.zip › Figure 2/Figure 2D/D3-siNC.jpg]

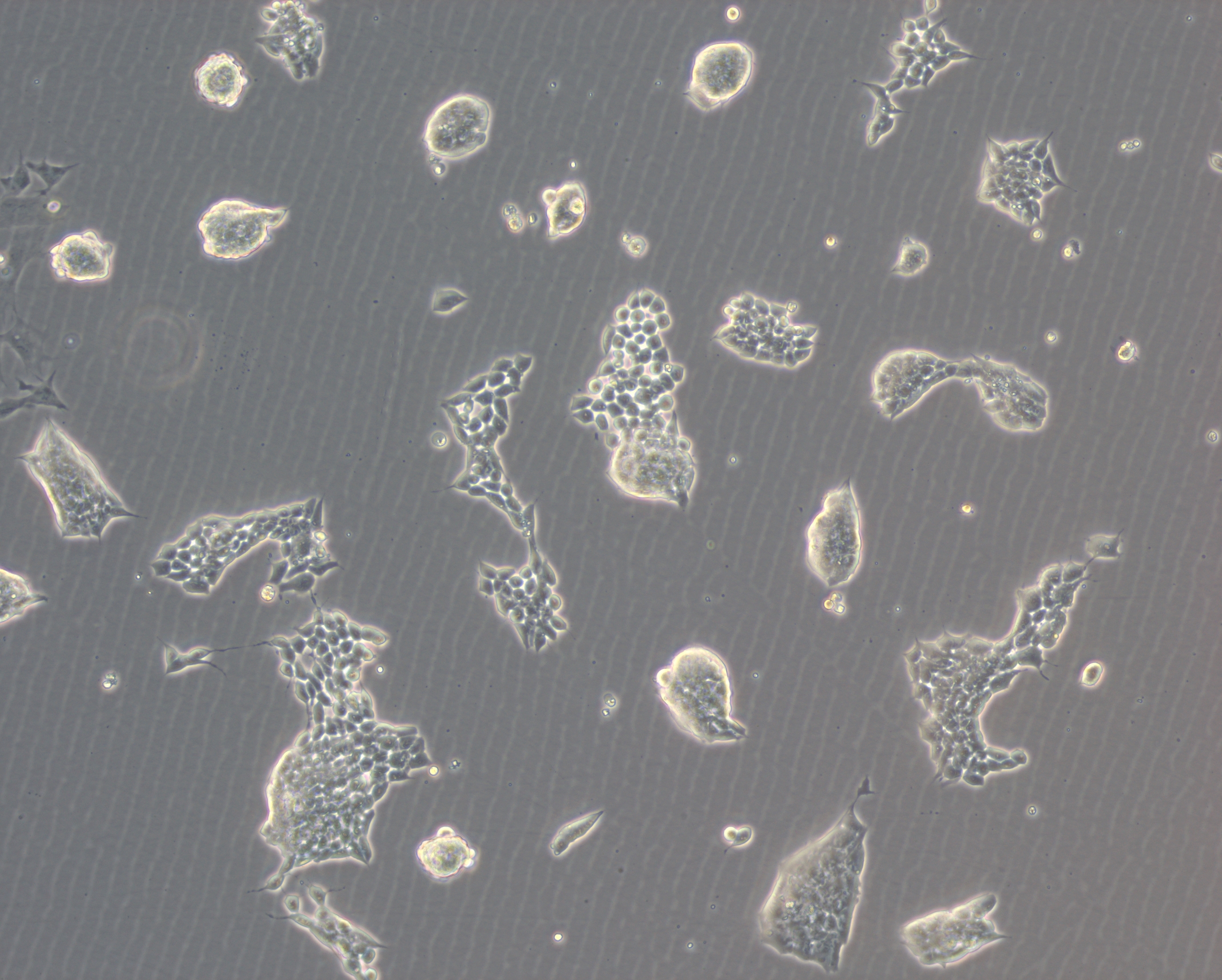

Supplement: Supplementary file 13 — Source data Fig. 2 [file 44318_2024_86_MOESM13_ESM.zip › Figure 2/Figure 2D/D3-siNr5a2.jpg]

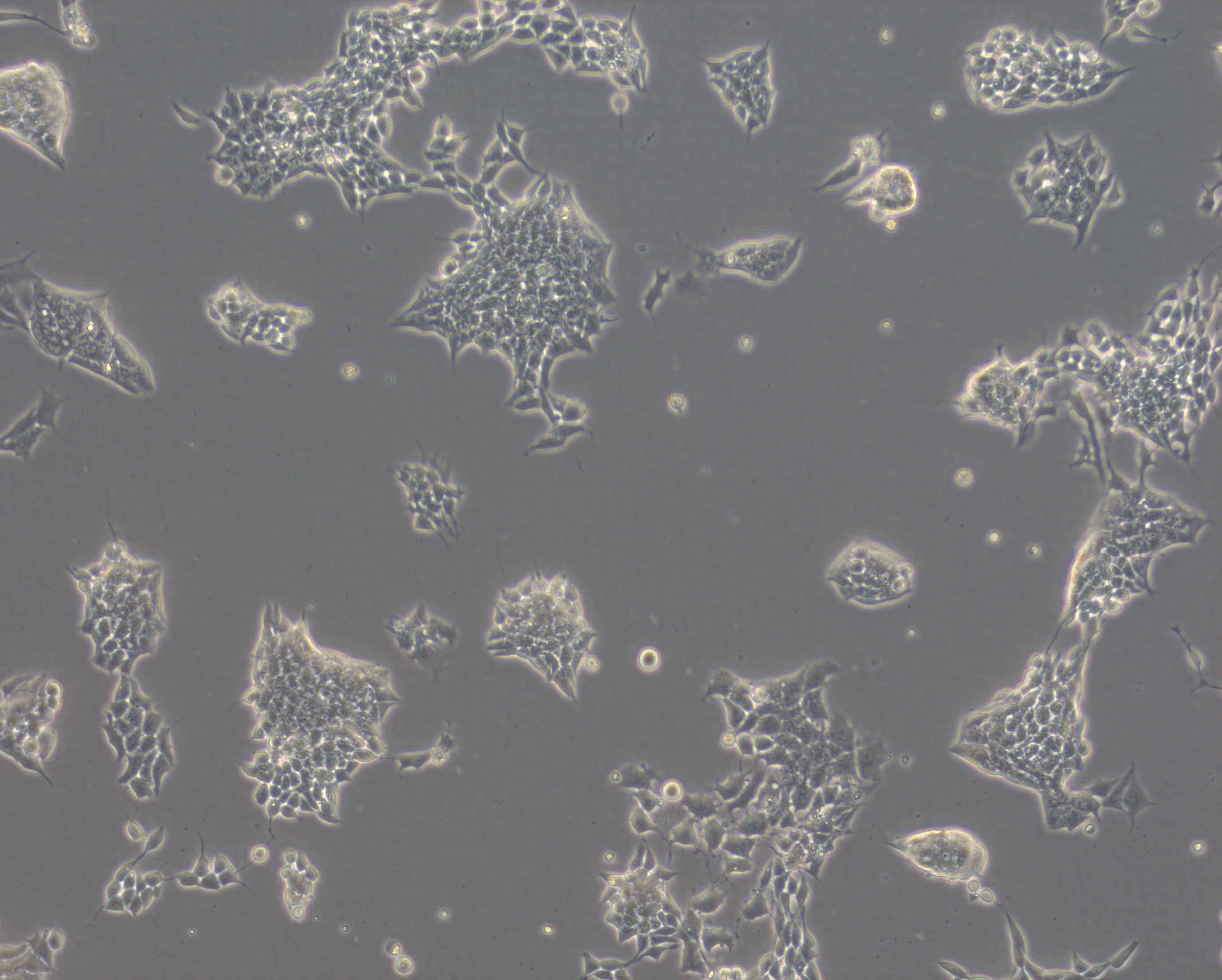

Supplement: Supplementary file 13 — Source data Fig. 2 [file 44318_2024_86_MOESM13_ESM.zip › Figure 2/Figure 2D/D3-siTead2.jpg]

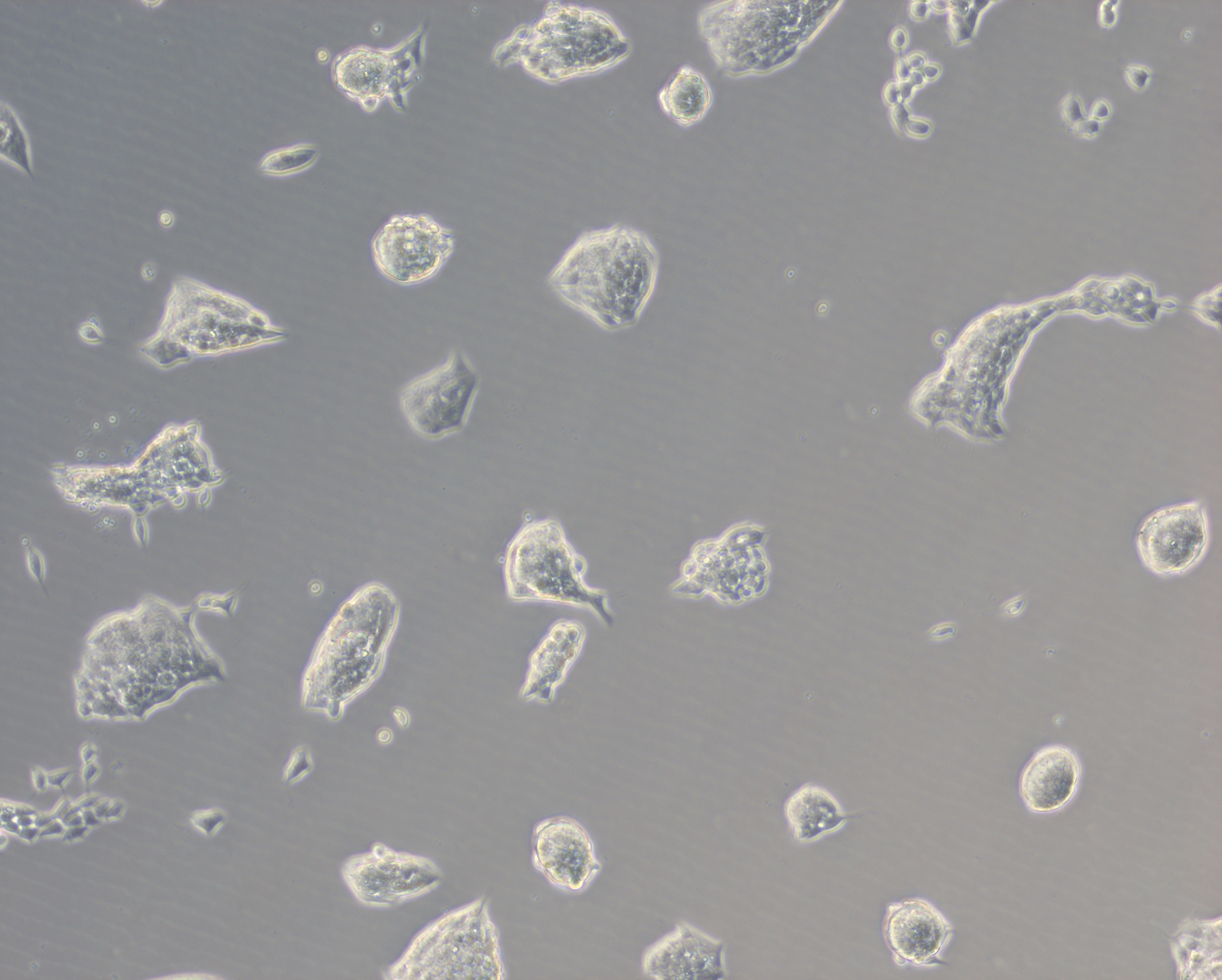

Supplement: Supplementary file 13 — Source data Fig. 2 [file 44318_2024_86_MOESM13_ESM.zip › Figure 2/Figure 2D/D3-siTead4.jpg]

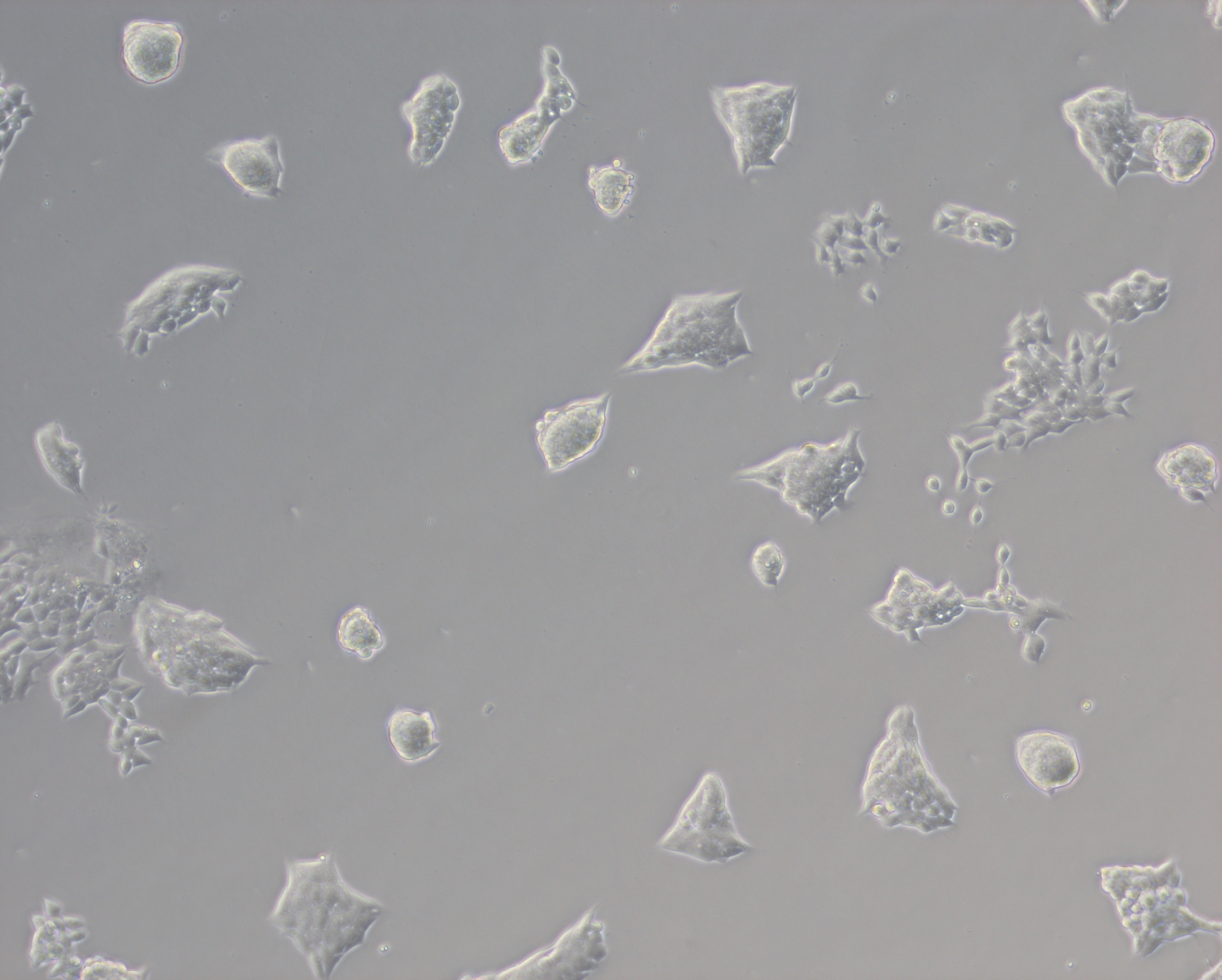

Supplement: Supplementary file 13 — Source data Fig. 2 [file 44318_2024_86_MOESM13_ESM.zip › Figure 2/Figure 2D/D3-siTfcp2l1.jpg]

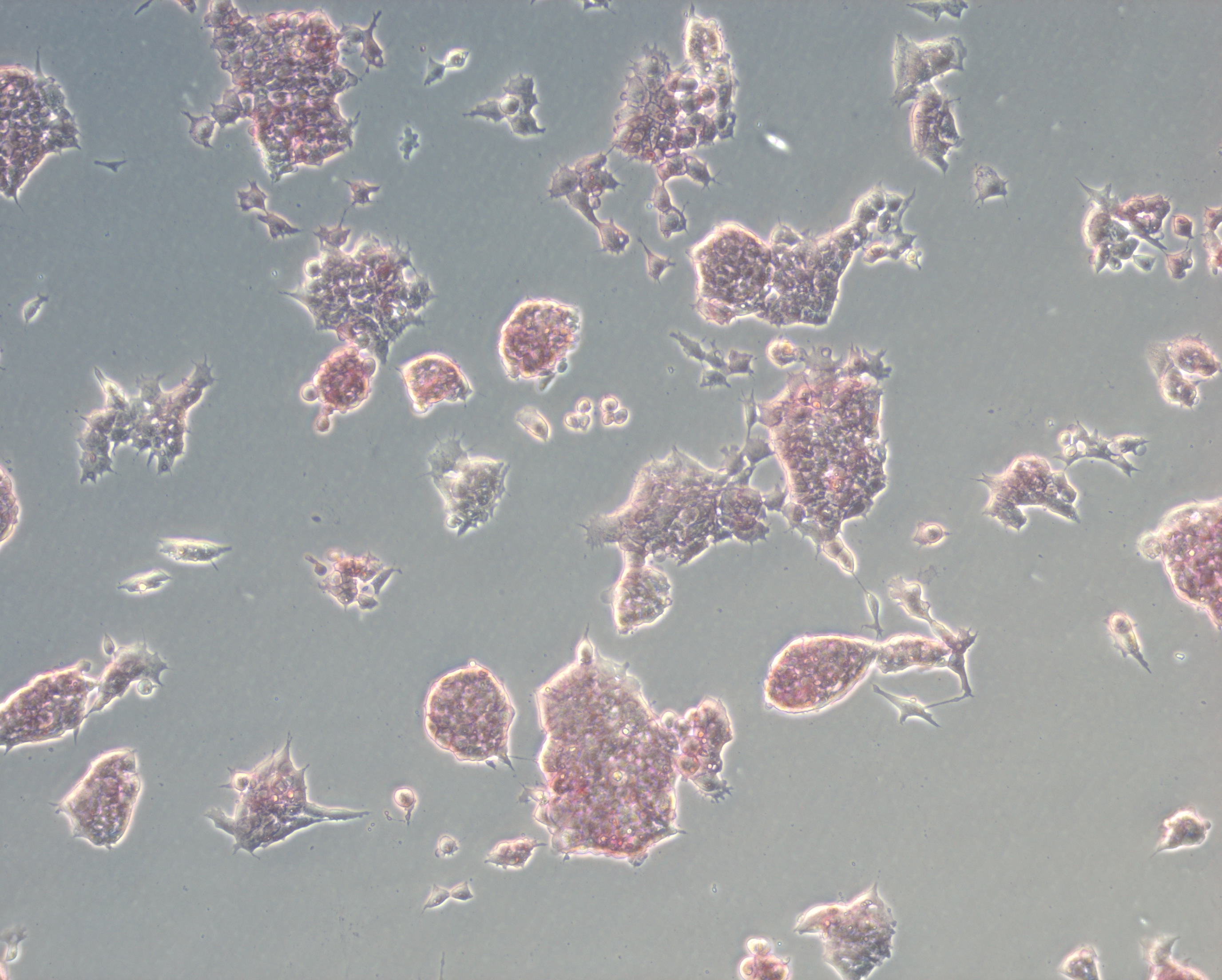

Supplement: Supplementary file 13 — Source data Fig. 2 [file 44318_2024_86_MOESM13_ESM.zip › Figure 2/Figure 2E/D0-siEsrrb.jpg]

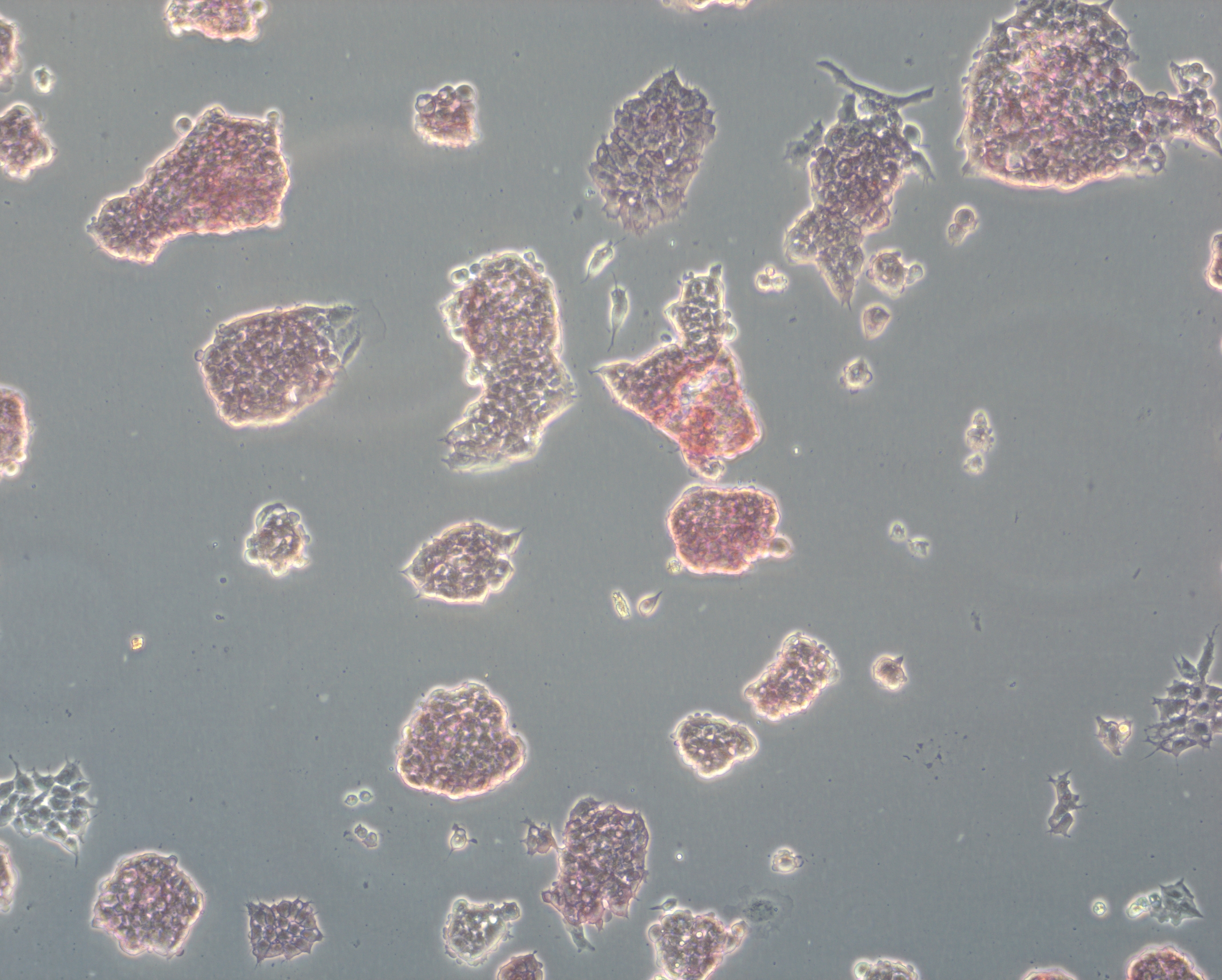

Supplement: Supplementary file 13 — Source data Fig. 2 [file 44318_2024_86_MOESM13_ESM.zip › Figure 2/Figure 2E/D0-siNC.jpg]

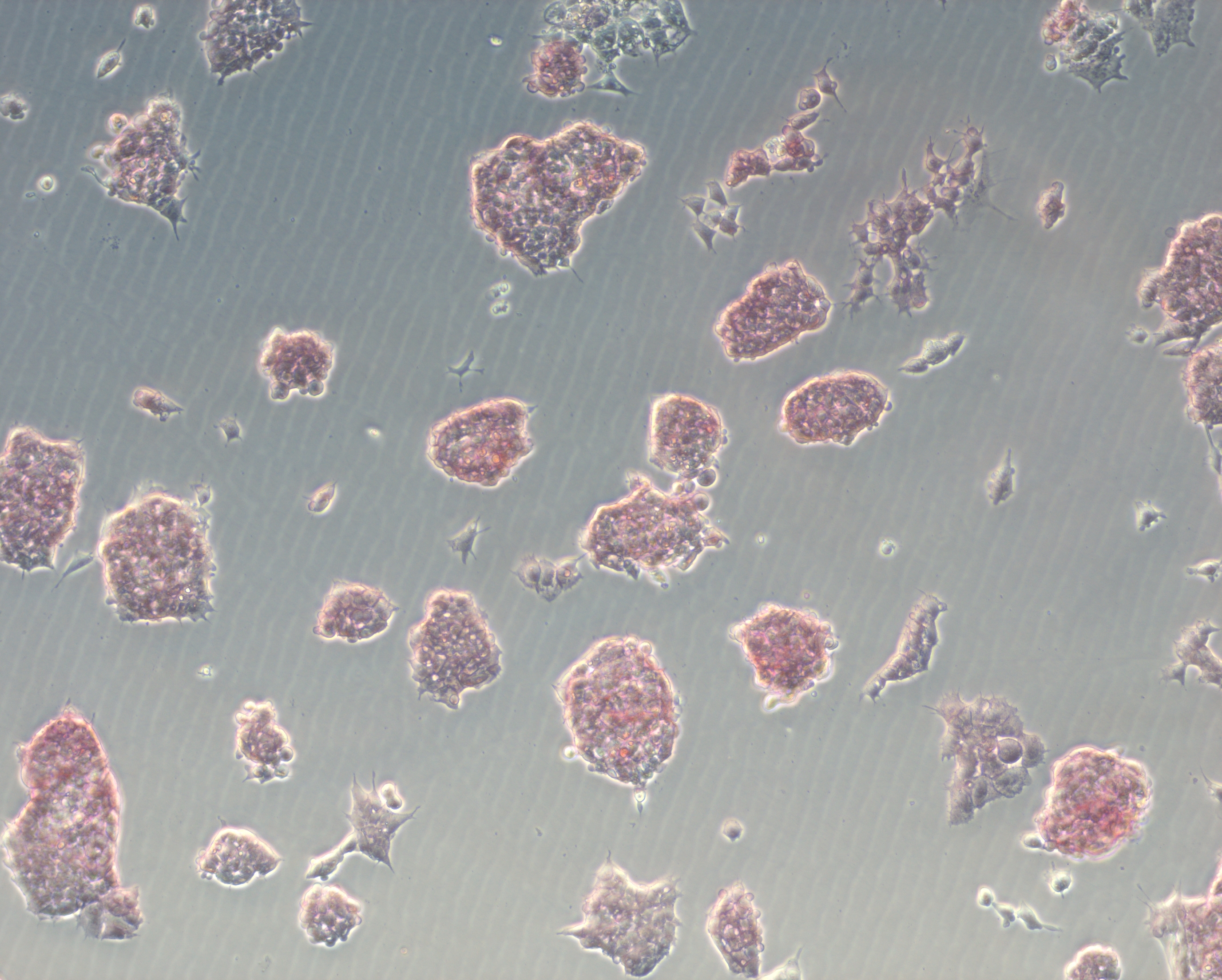

Supplement: Supplementary file 13 — Source data Fig. 2 [file 44318_2024_86_MOESM13_ESM.zip › Figure 2/Figure 2E/D0-siNr5a2.jpg]

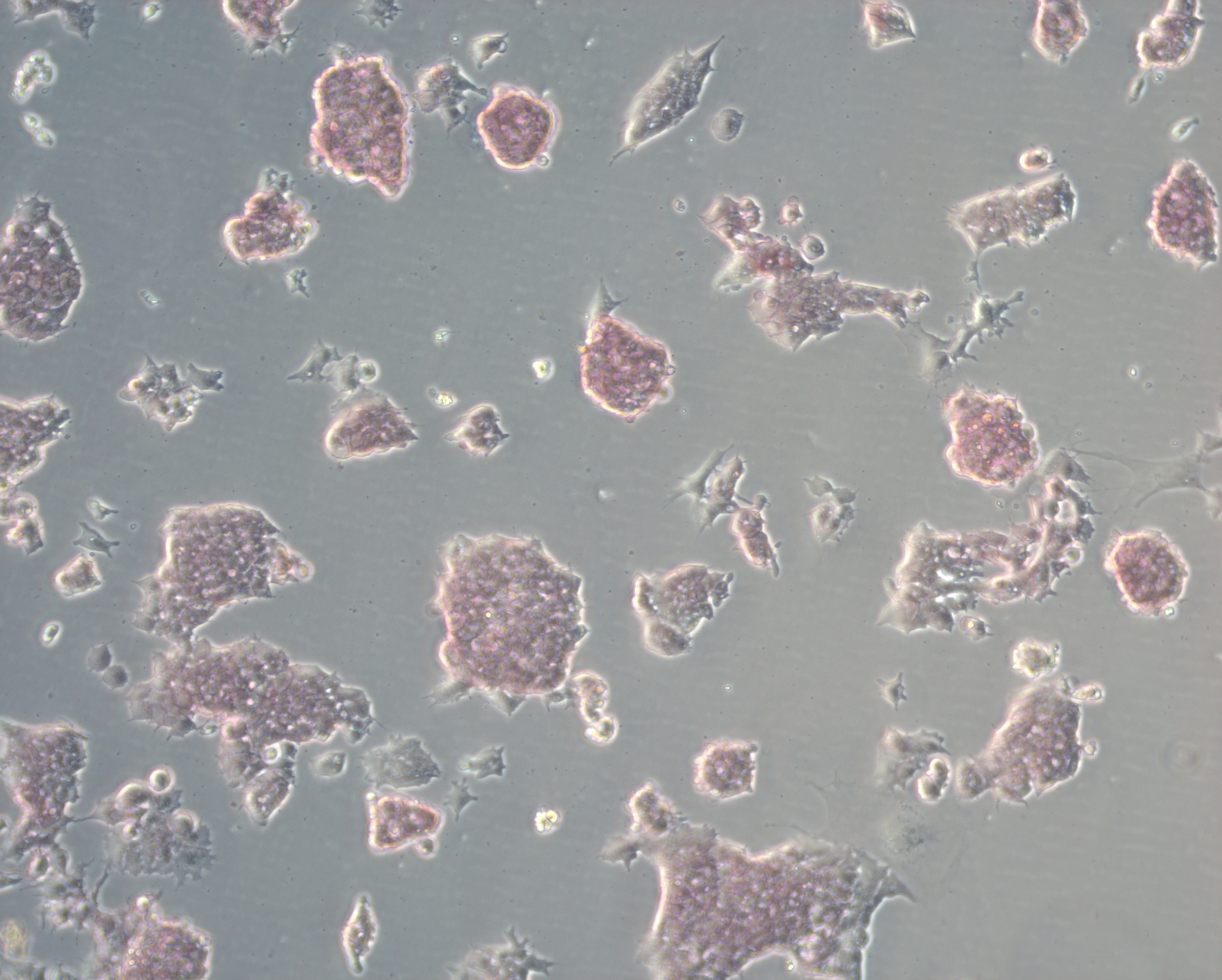

Supplement: Supplementary file 13 — Source data Fig. 2 [file 44318_2024_86_MOESM13_ESM.zip › Figure 2/Figure 2E/D0-siTead2.jpg]

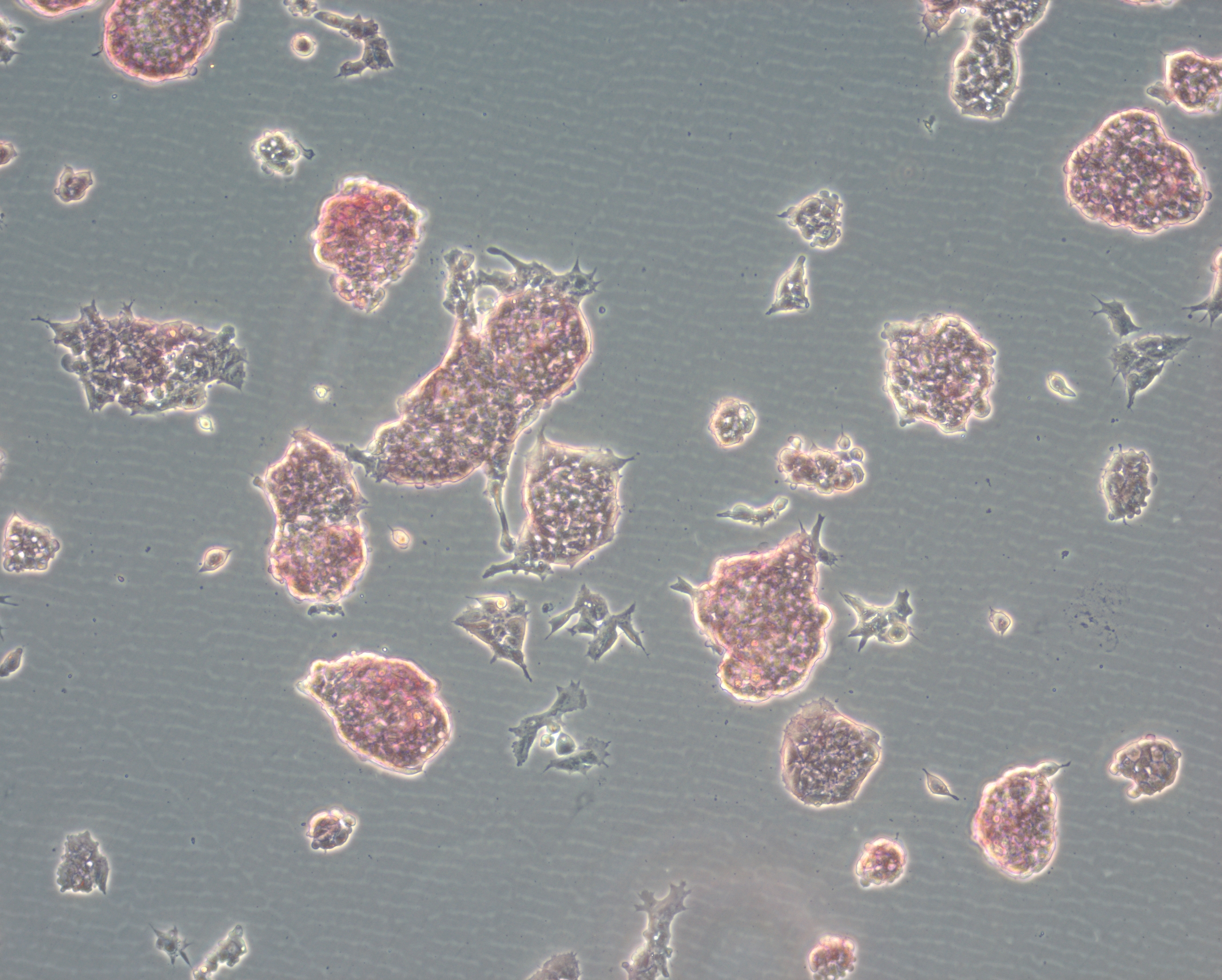

Supplement: Supplementary file 13 — Source data Fig. 2 [file 44318_2024_86_MOESM13_ESM.zip › Figure 2/Figure 2E/D0-siTead4.jpg]

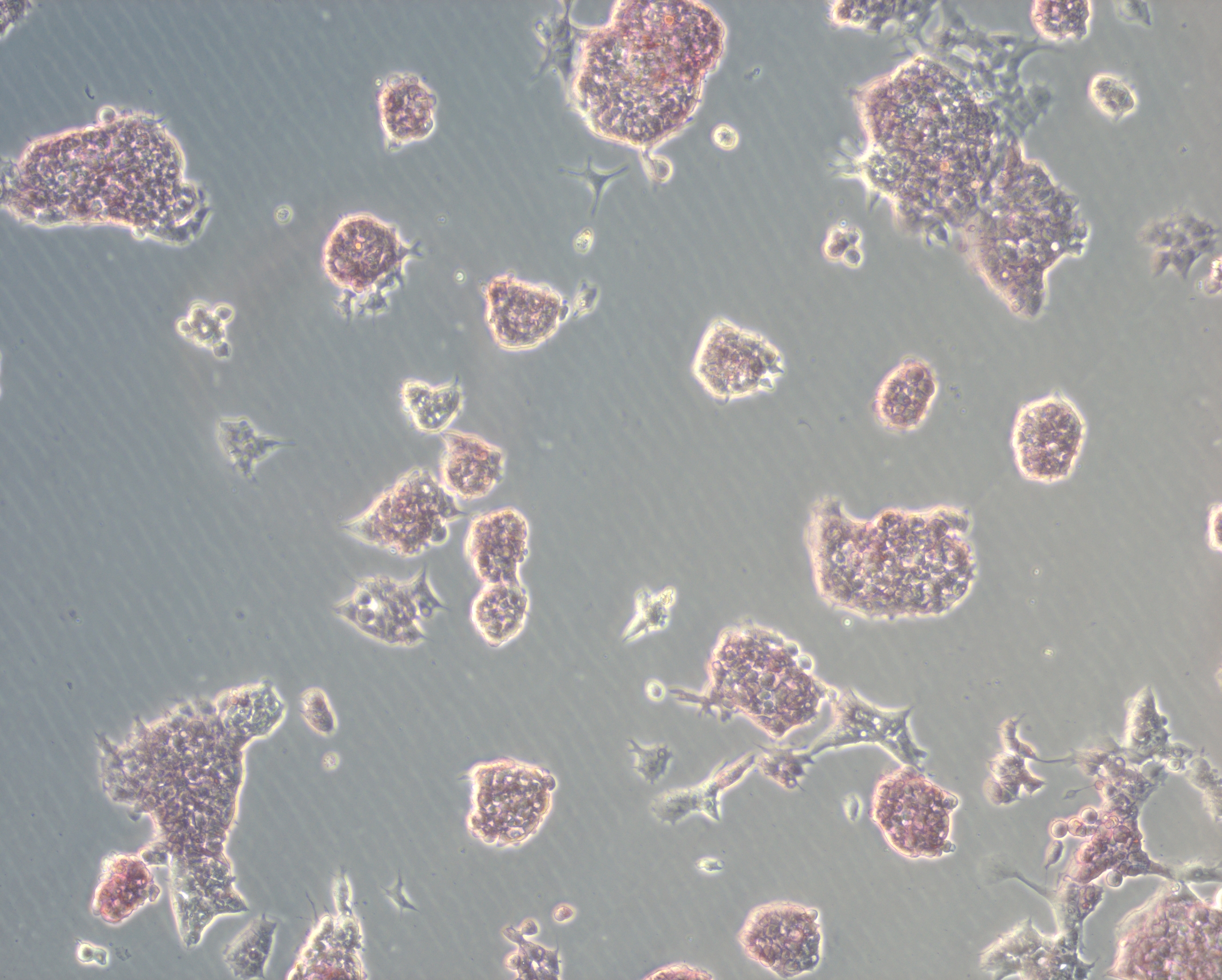

Supplement: Supplementary file 13 — Source data Fig. 2 [file 44318_2024_86_MOESM13_ESM.zip › Figure 2/Figure 2E/D0-siTfcp2l1.jpg]

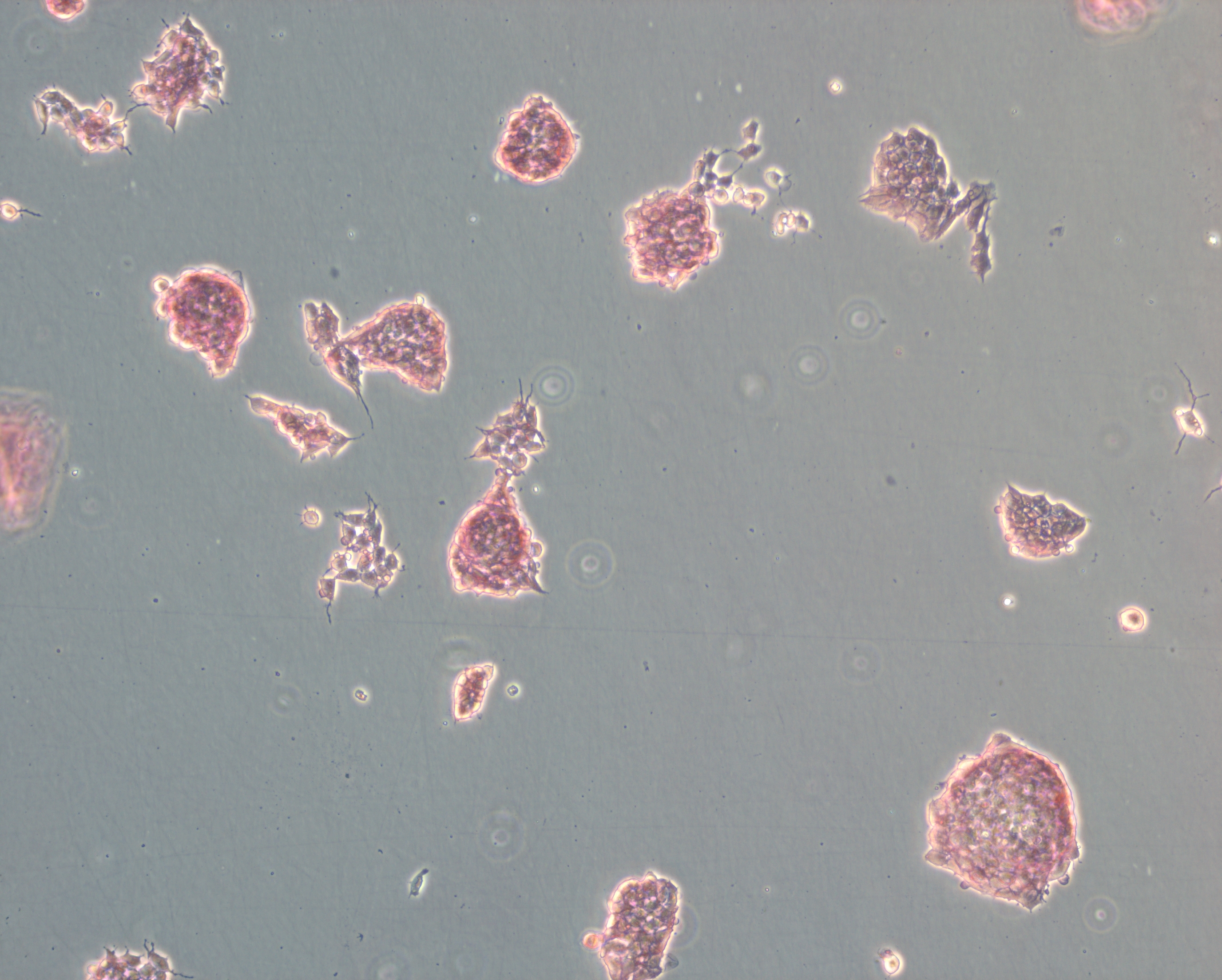

Supplement: Supplementary file 13 — Source data Fig. 2 [file 44318_2024_86_MOESM13_ESM.zip › Figure 2/Figure 2E/D3-siErssb.jpg]

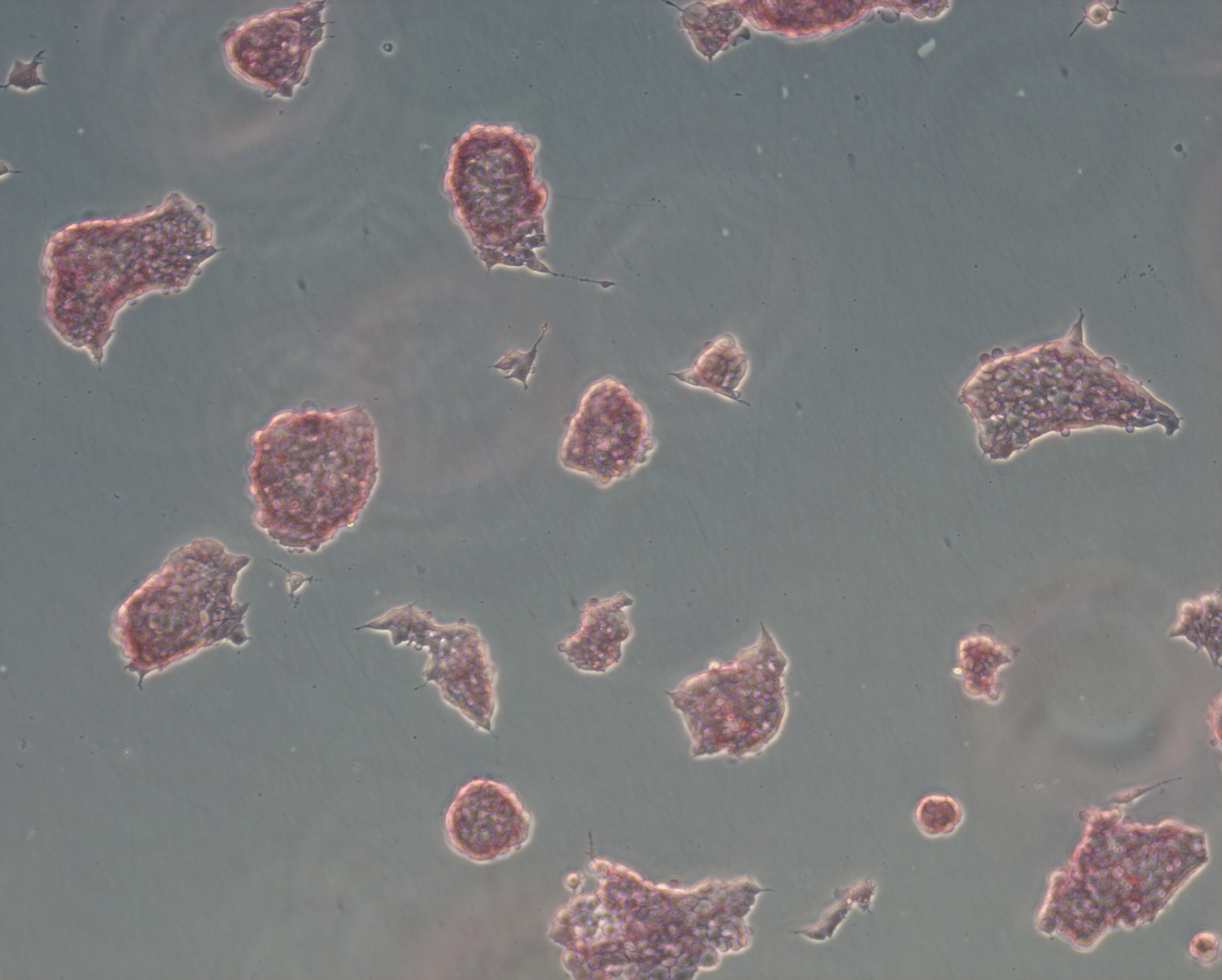

Supplement: Supplementary file 13 — Source data Fig. 2 [file 44318_2024_86_MOESM13_ESM.zip › Figure 2/Figure 2E/D3-siNC.jpg]

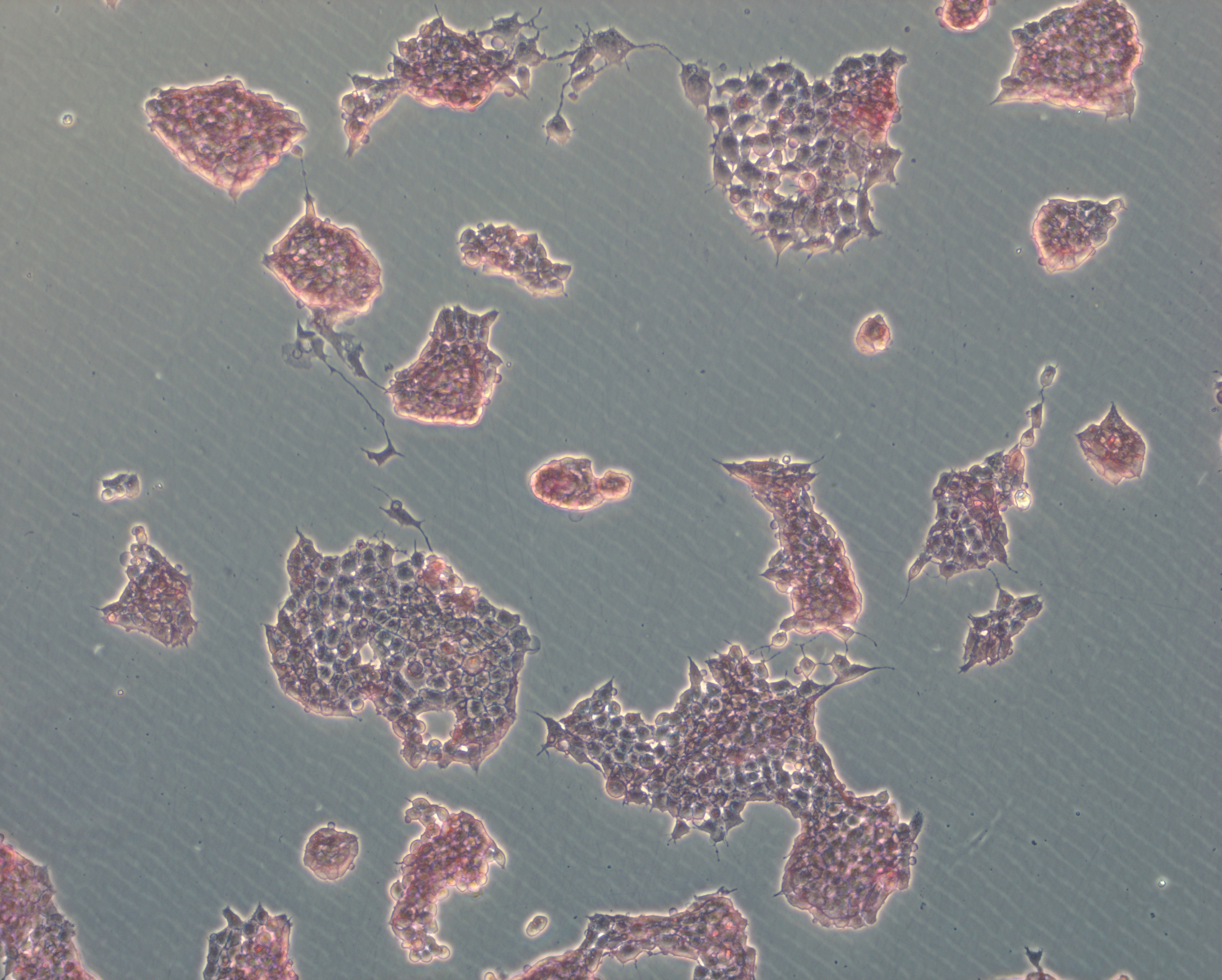

Supplement: Supplementary file 13 — Source data Fig. 2 [file 44318_2024_86_MOESM13_ESM.zip › Figure 2/Figure 2E/D3-siNr5a2.jpg]

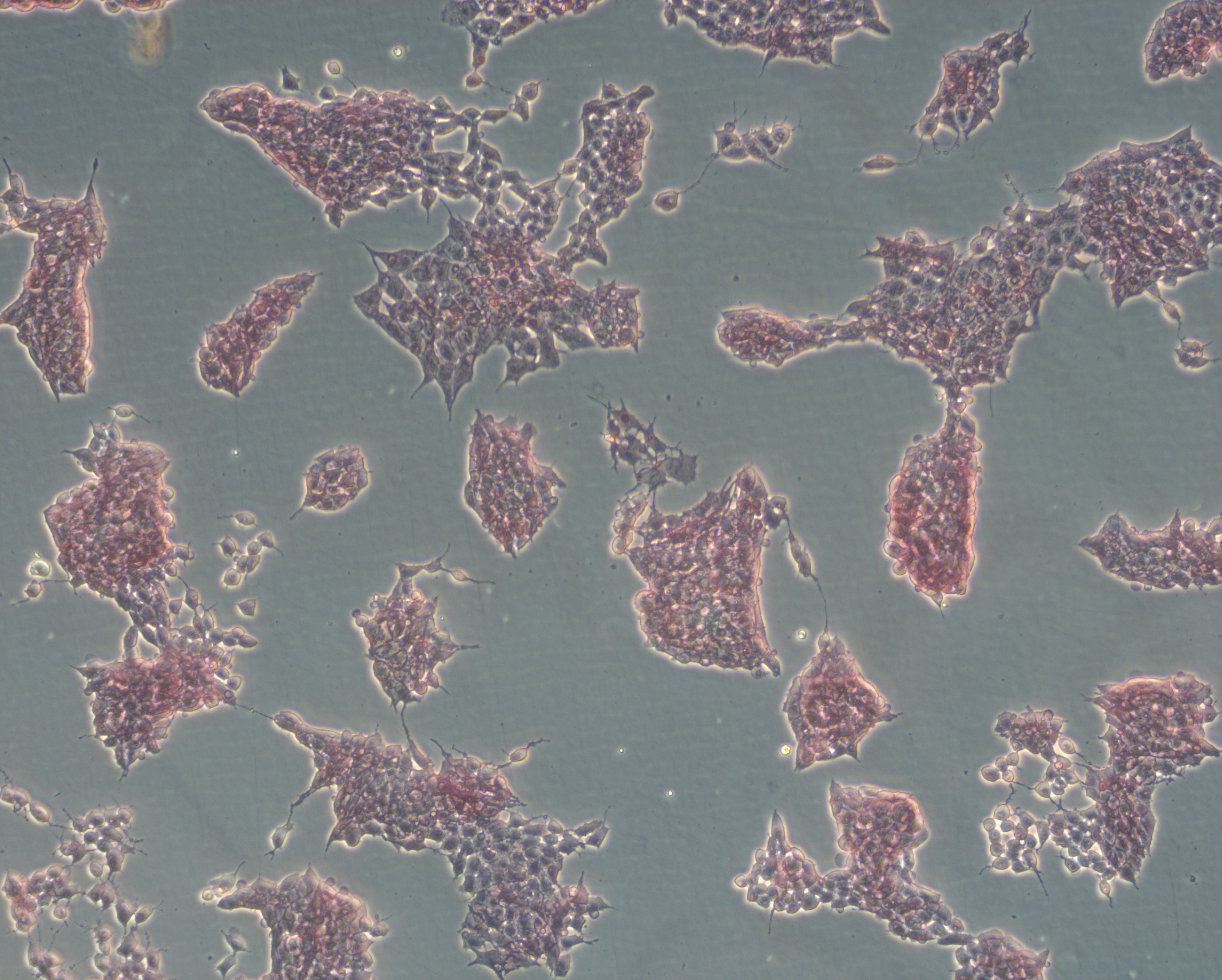

Supplement: Supplementary file 13 — Source data Fig. 2 [file 44318_2024_86_MOESM13_ESM.zip › Figure 2/Figure 2E/D3-siTead2.jpg]

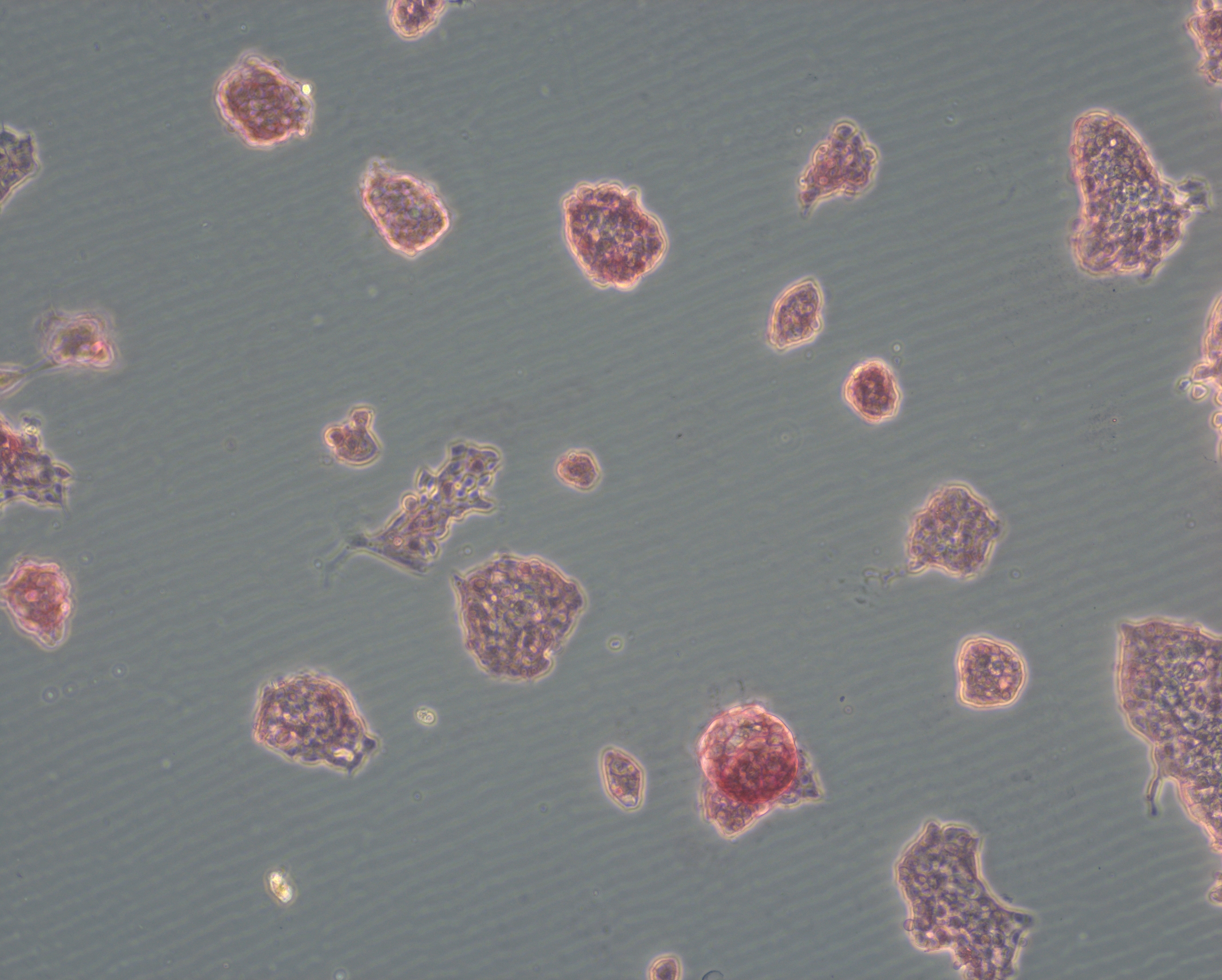

Supplement: Supplementary file 13 — Source data Fig. 2 [file 44318_2024_86_MOESM13_ESM.zip › Figure 2/Figure 2E/D3-siTead4.jpg]

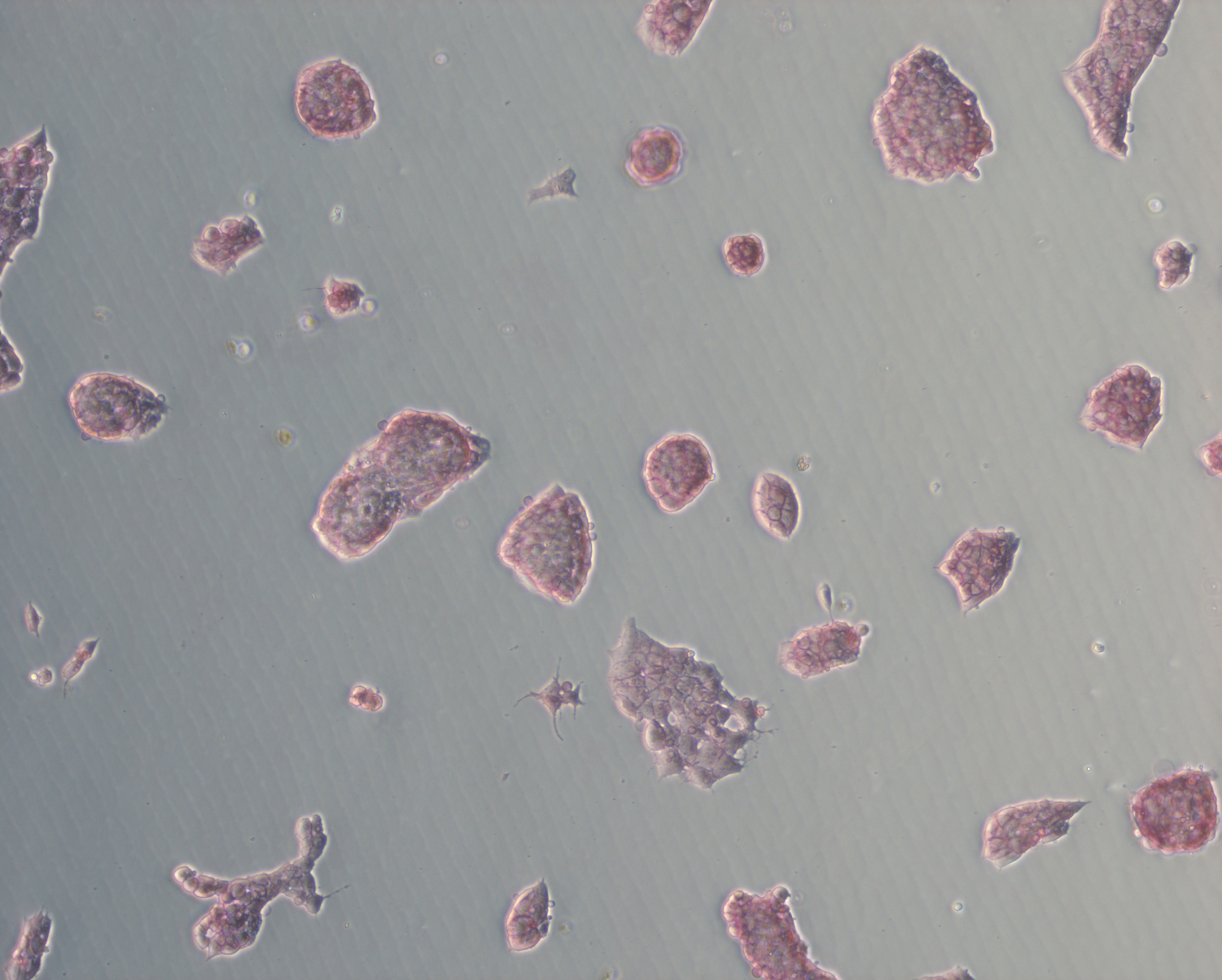

Supplement: Supplementary file 13 — Source data Fig. 2 [file 44318_2024_86_MOESM13_ESM.zip › Figure 2/Figure 2E/D3-siTfcp2l1.jpg]

Figure 3A

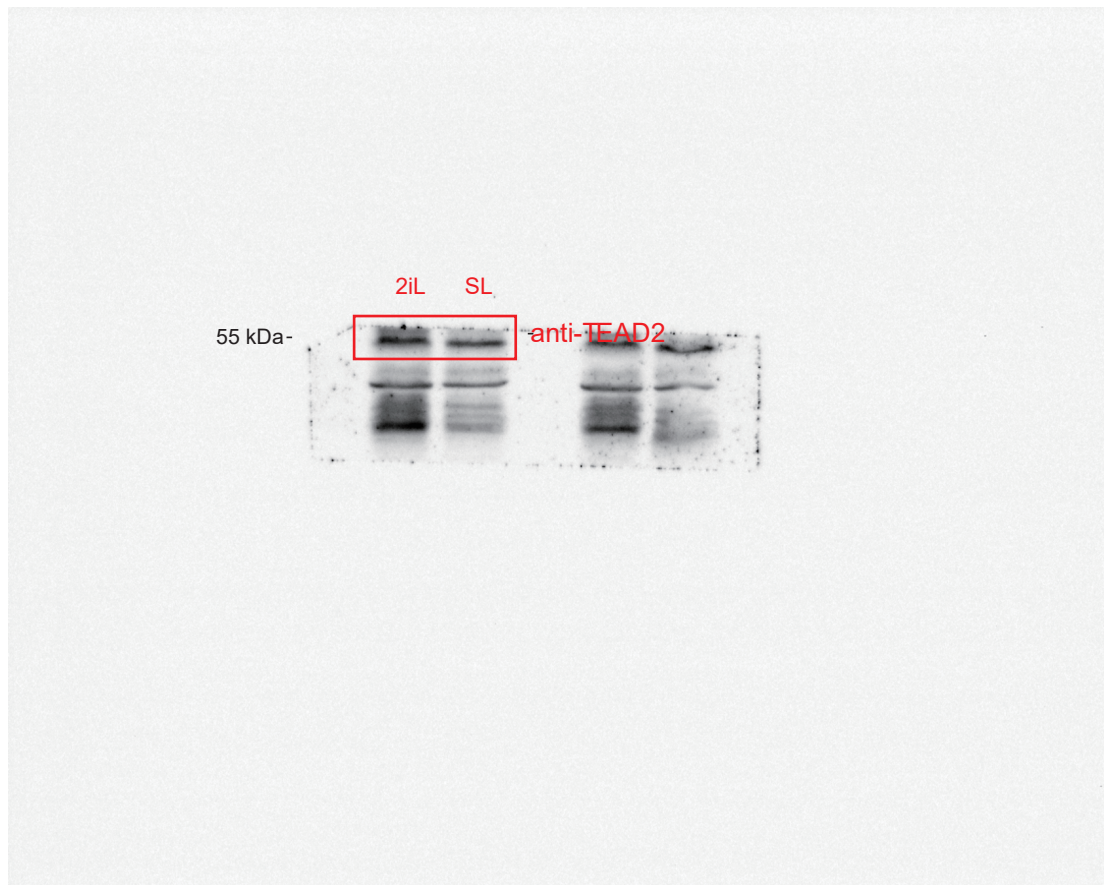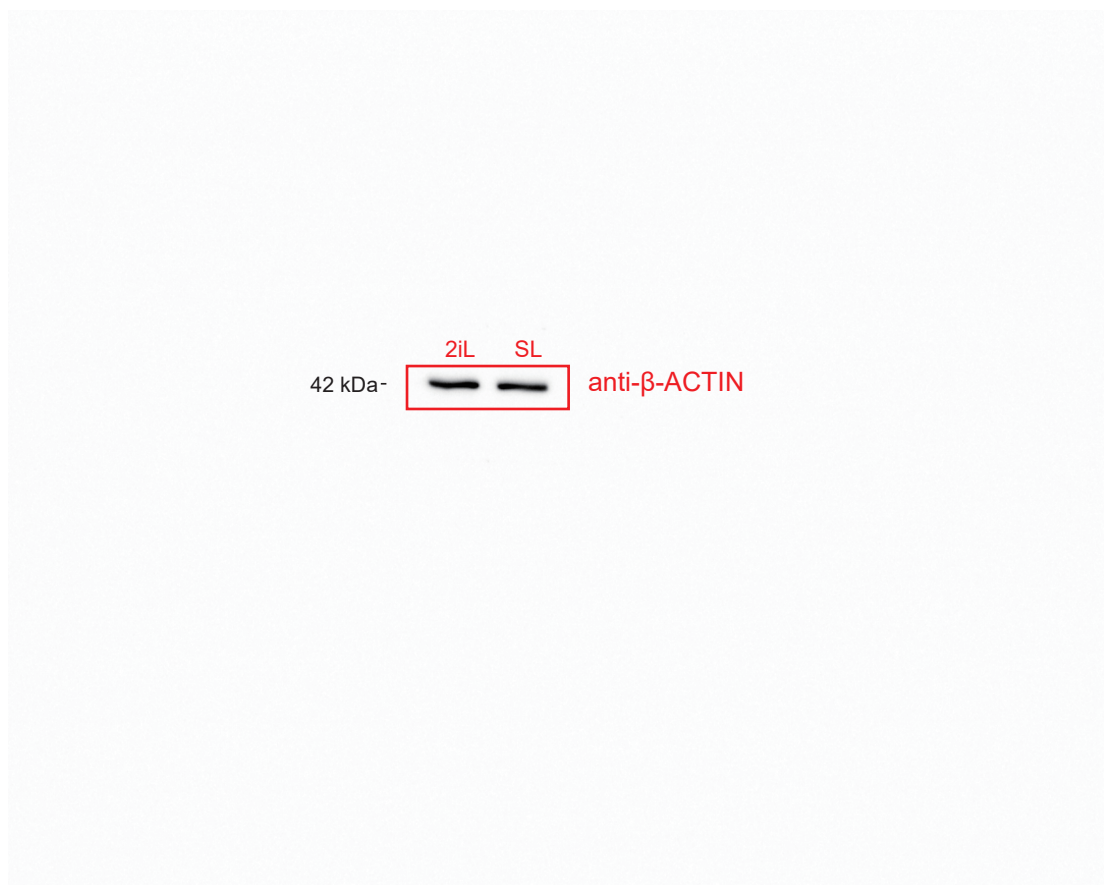

Supplement: Supplementary file 14 — Source data Fig. 3 [file 44318_2024_86_MOESM14_ESM.zip › Figure 3/Figure 3A/Western bolt-TEAD2 + ACTIN.pdf]

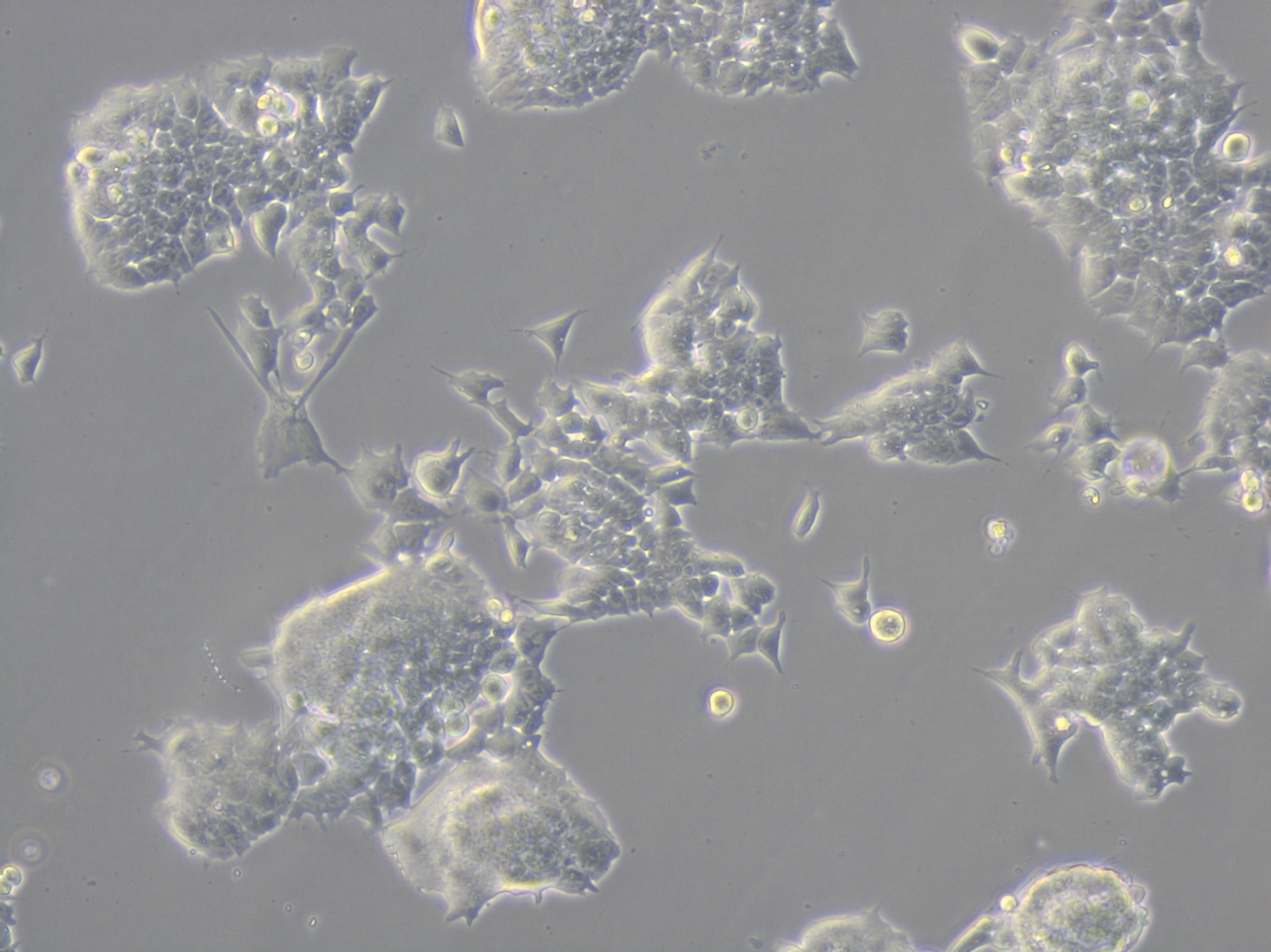

Supplement: Supplementary file 14 — Source data Fig. 3 [file 44318_2024_86_MOESM14_ESM.zip › Figure 3/Figure 3C/D0-Tead2+- 1#.jpg]

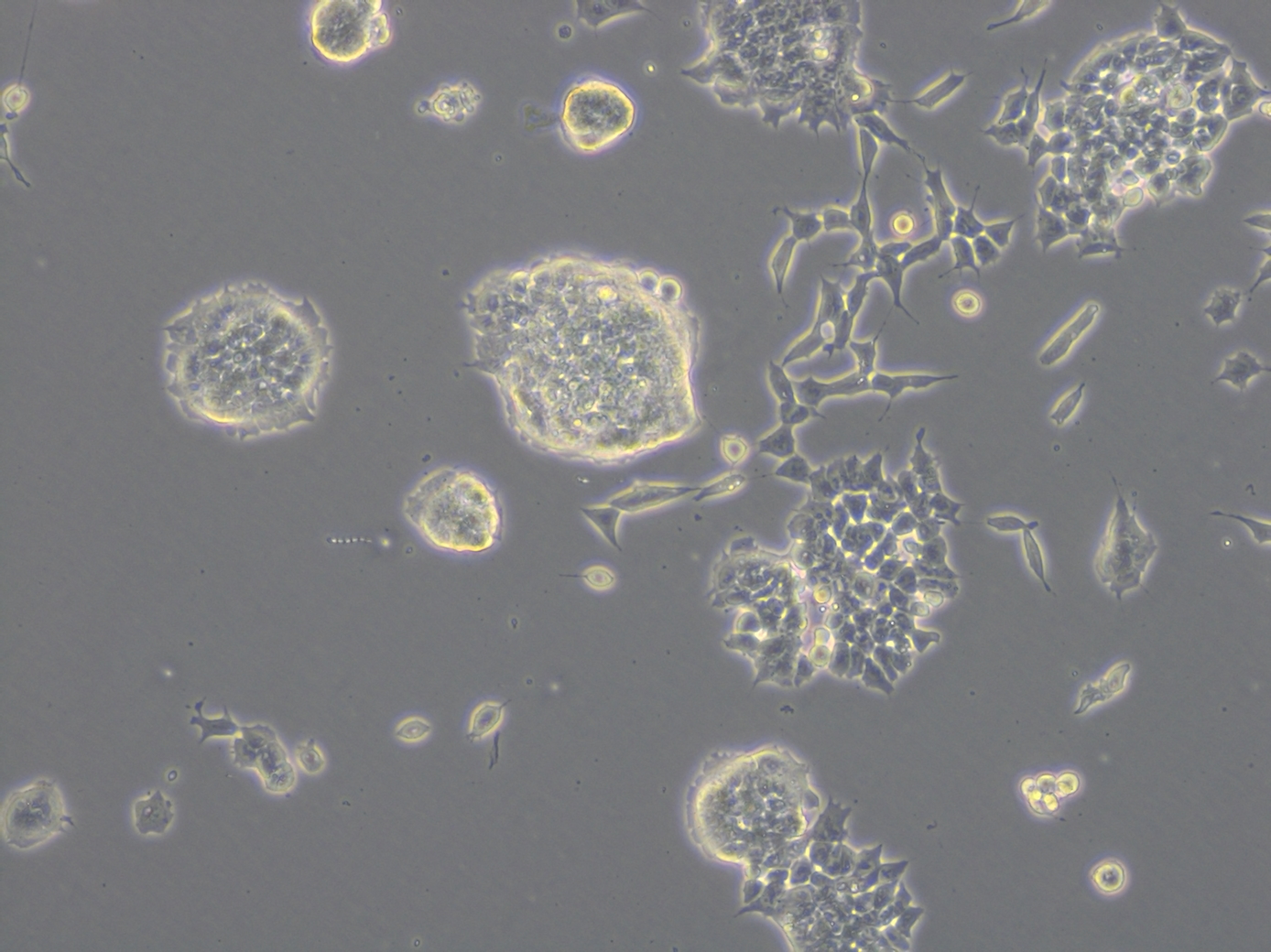

Supplement: Supplementary file 14 — Source data Fig. 3 [file 44318_2024_86_MOESM14_ESM.zip › Figure 3/Figure 3C/D0-Tead2+- 2#.jpg]

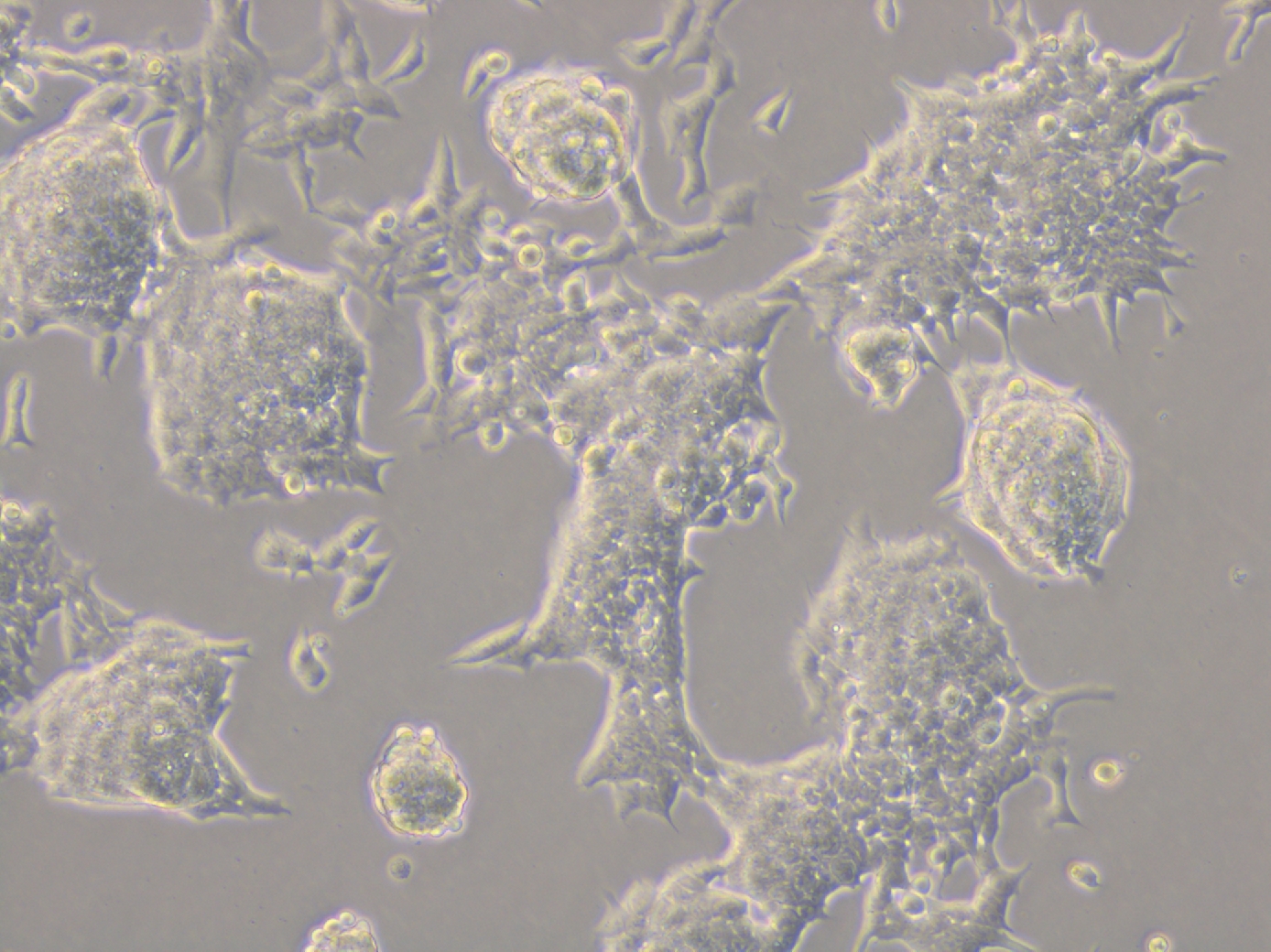

Supplement: Supplementary file 14 — Source data Fig. 3 [file 44318_2024_86_MOESM14_ESM.zip › Figure 3/Figure 3C/D0-Tead2-- 1#.jpg]

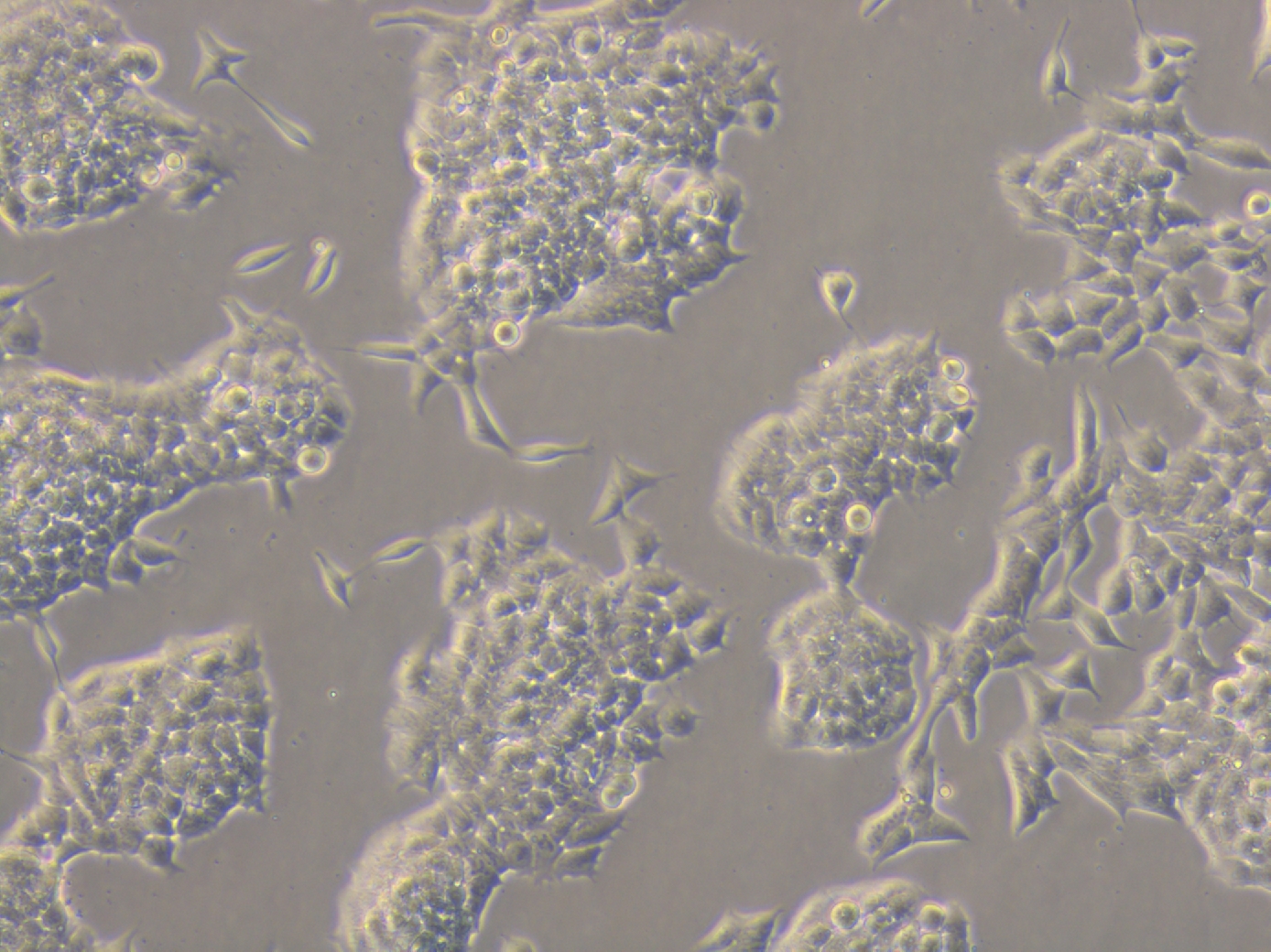

Supplement: Supplementary file 14 — Source data Fig. 3 [file 44318_2024_86_MOESM14_ESM.zip › Figure 3/Figure 3C/D0-Tead2-- 2#.jpg]

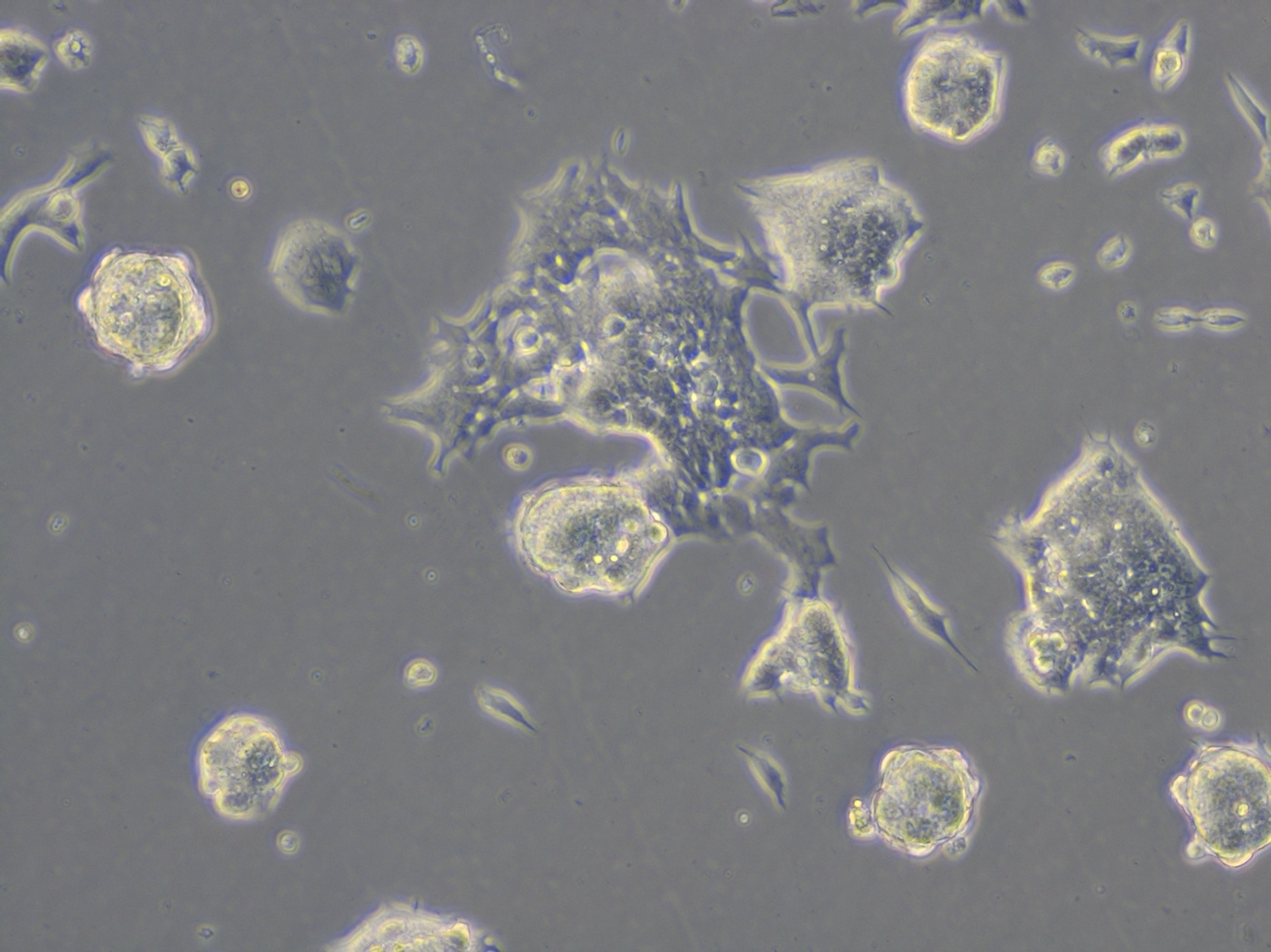

Supplement: Supplementary file 14 — Source data Fig. 3 [file 44318_2024_86_MOESM14_ESM.zip › Figure 3/Figure 3C/D0-WT.jpg]

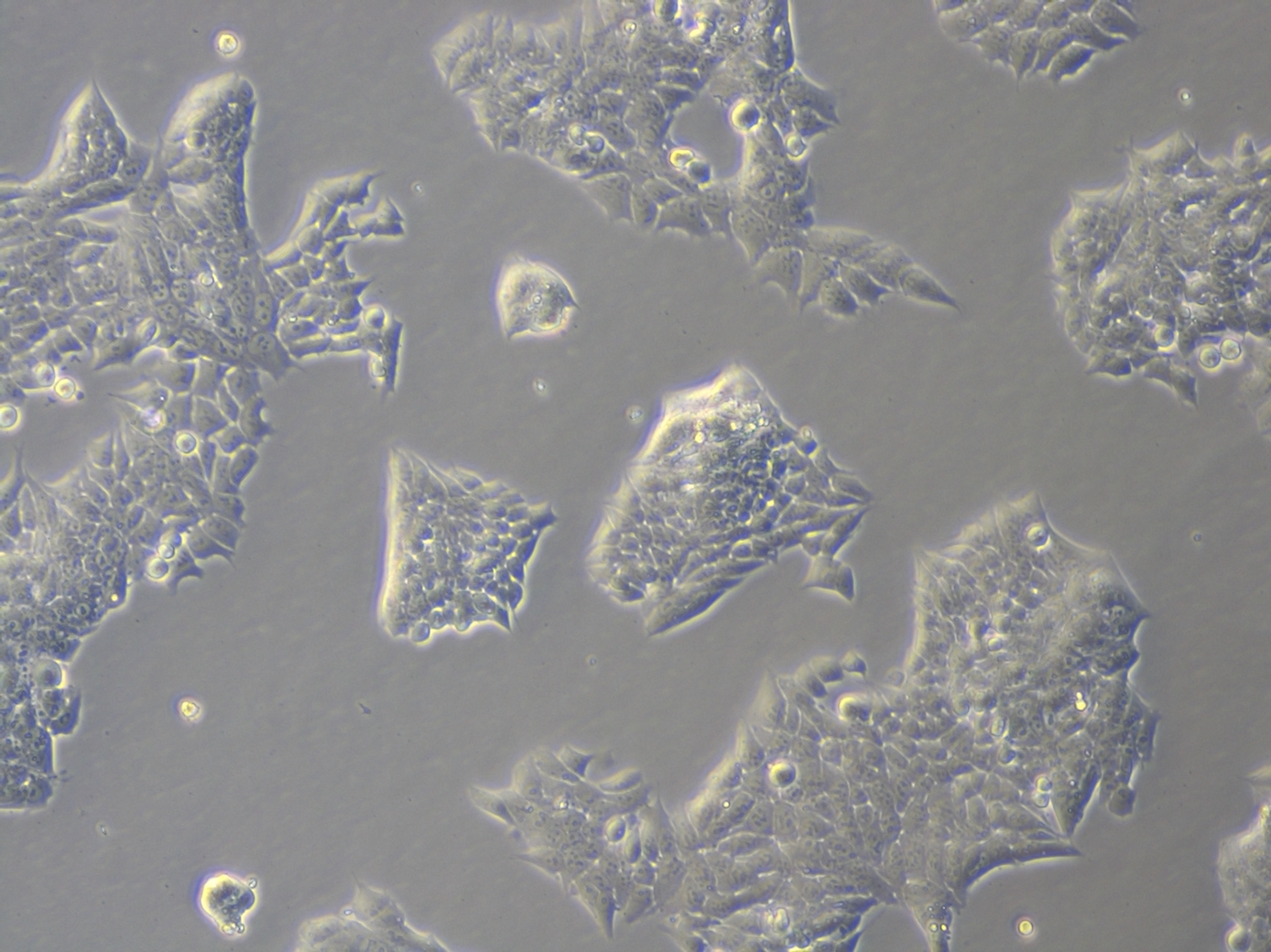

Supplement: Supplementary file 14 — Source data Fig. 3 [file 44318_2024_86_MOESM14_ESM.zip › Figure 3/Figure 3C/D3-Tead2+- 1#.jpg]

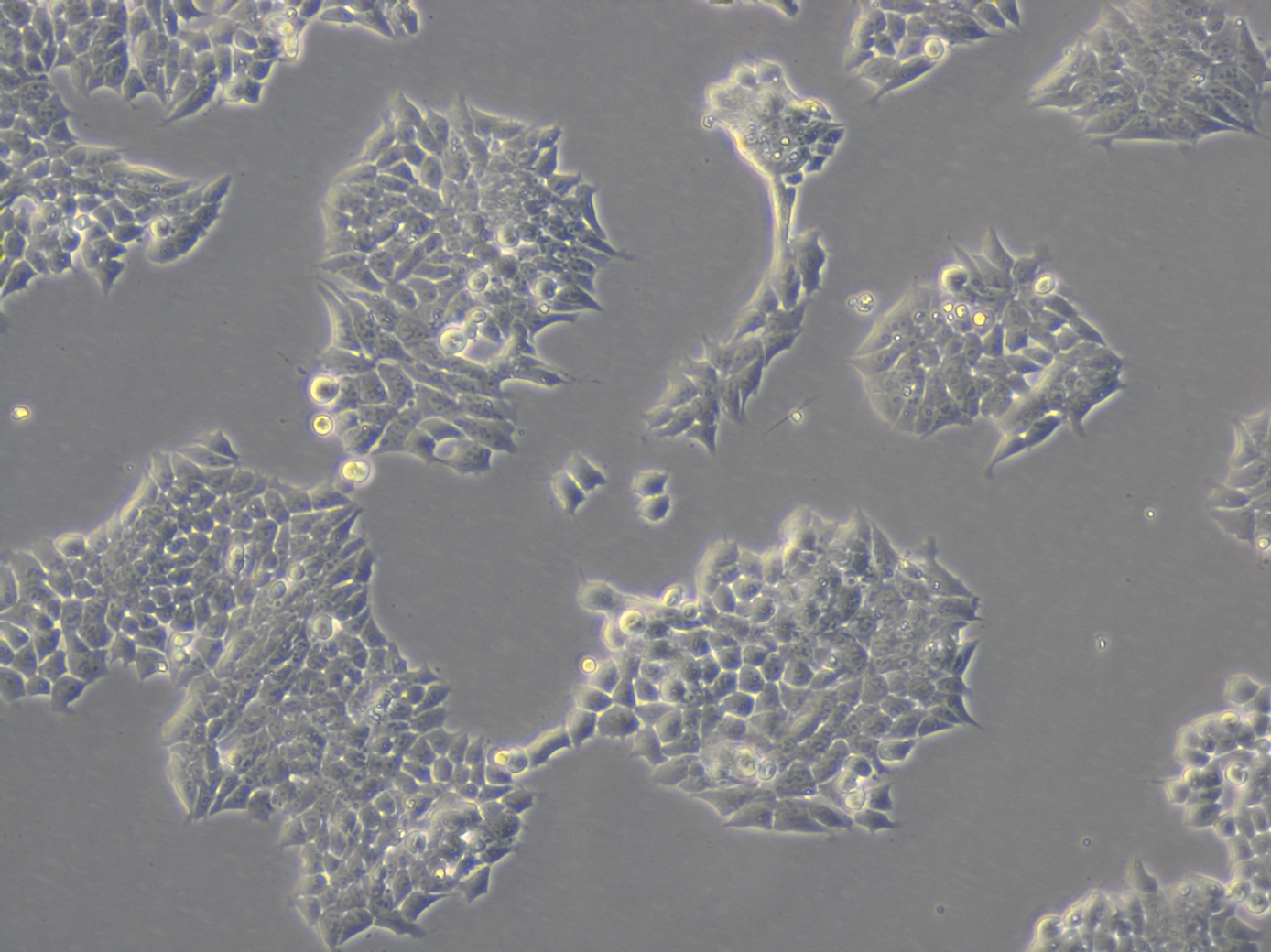

Supplement: Supplementary file 14 — Source data Fig. 3 [file 44318_2024_86_MOESM14_ESM.zip › Figure 3/Figure 3C/D3-Tead2+- 2#.jpg]

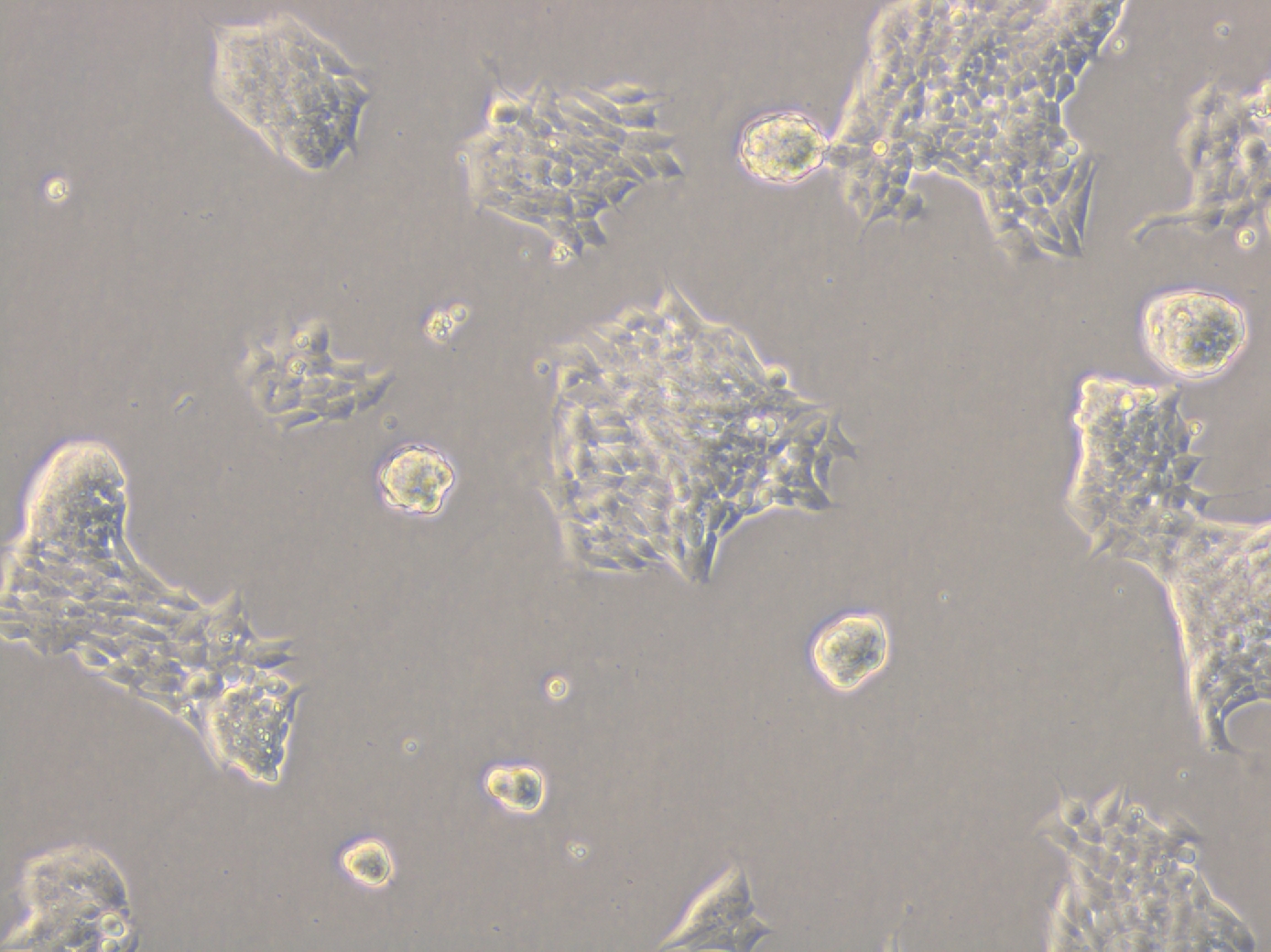

Supplement: Supplementary file 14 — Source data Fig. 3 [file 44318_2024_86_MOESM14_ESM.zip › Figure 3/Figure 3C/D3-Tead2-- 1#.jpg]

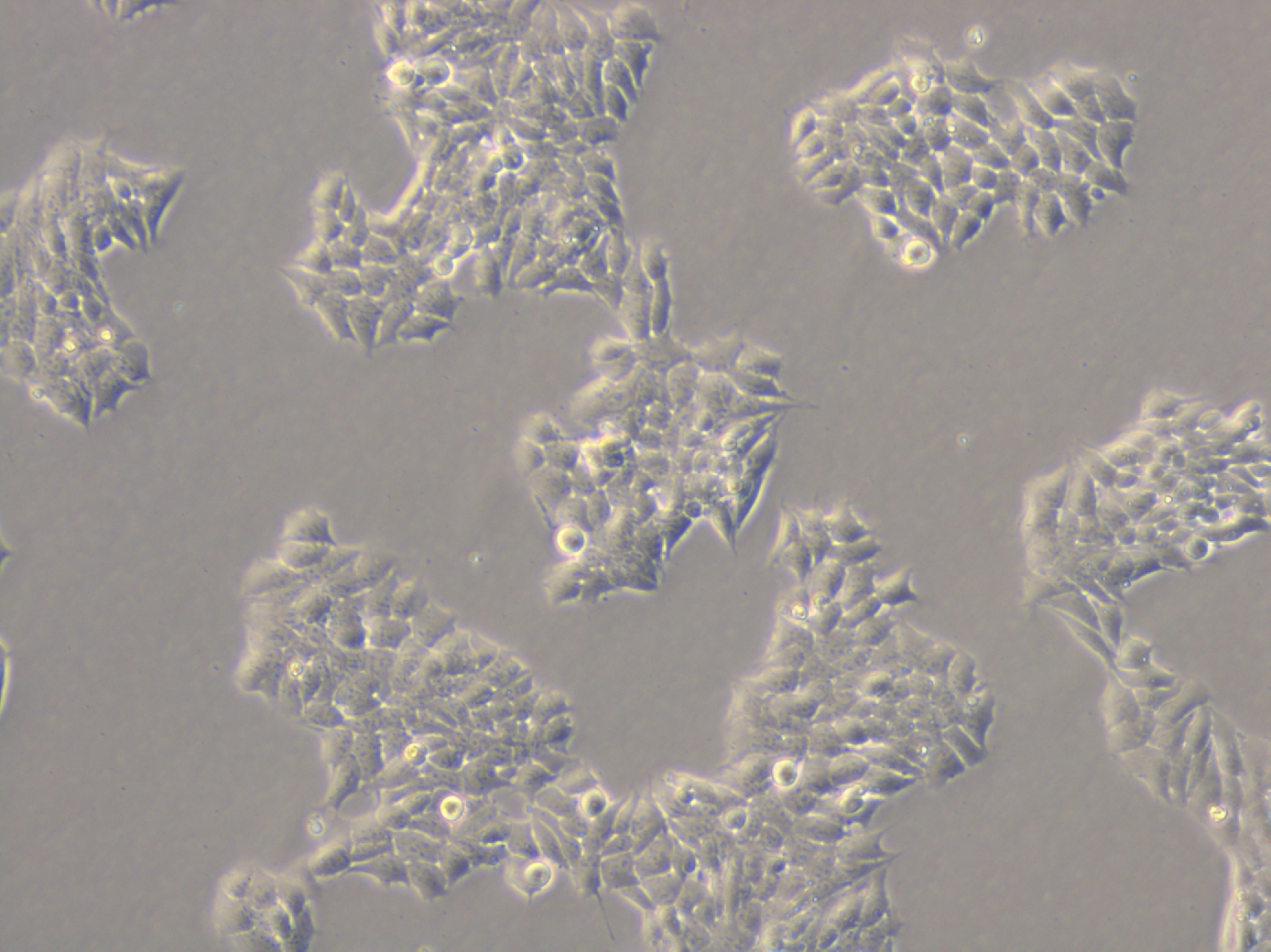

Supplement: Supplementary file 14 — Source data Fig. 3 [file 44318_2024_86_MOESM14_ESM.zip › Figure 3/Figure 3C/D3-Tead2-- 2#.jpg]

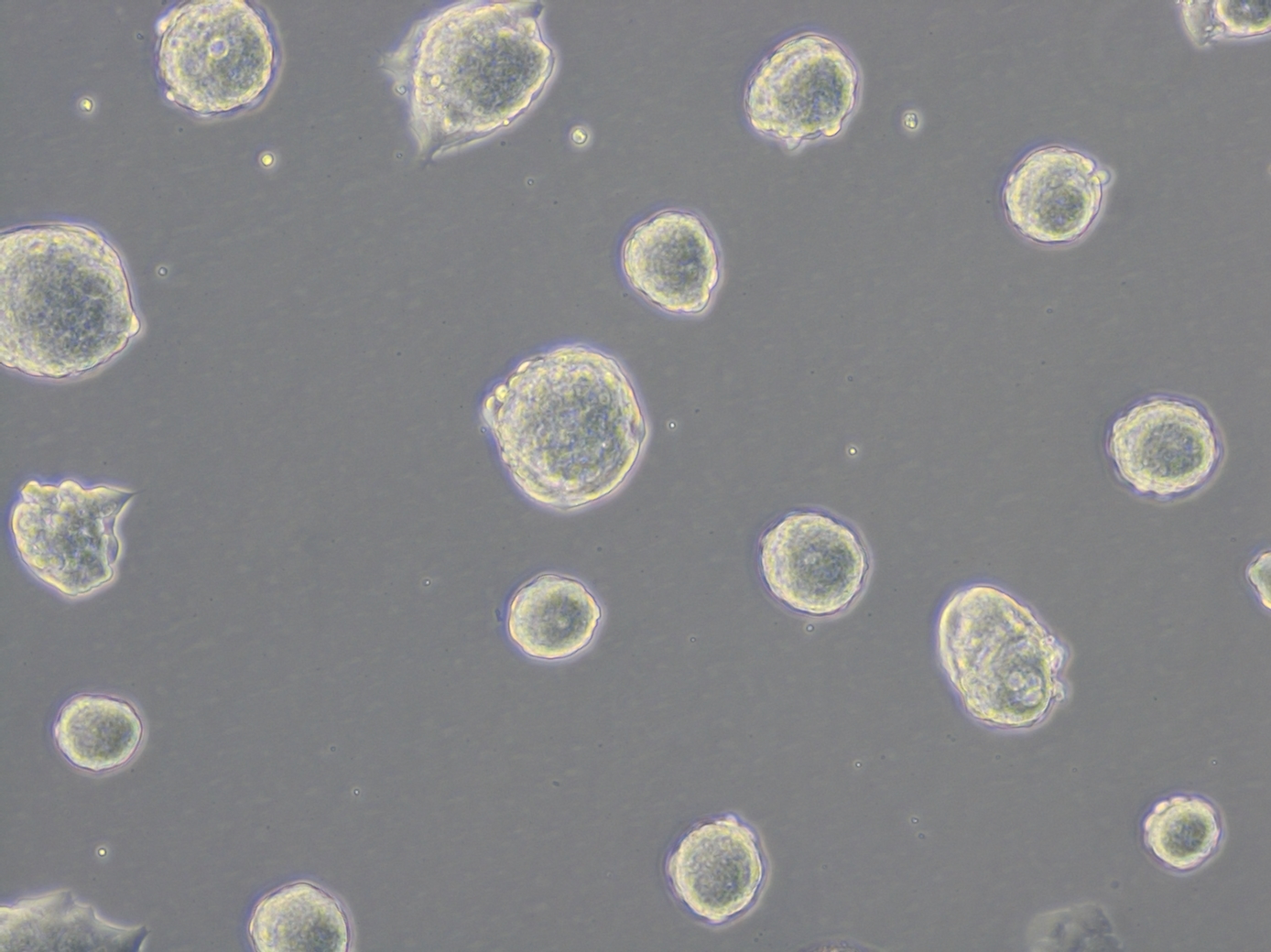

Supplement: Supplementary file 14 — Source data Fig. 3 [file 44318_2024_86_MOESM14_ESM.zip › Figure 3/Figure 3C/D3-WT.jpg]

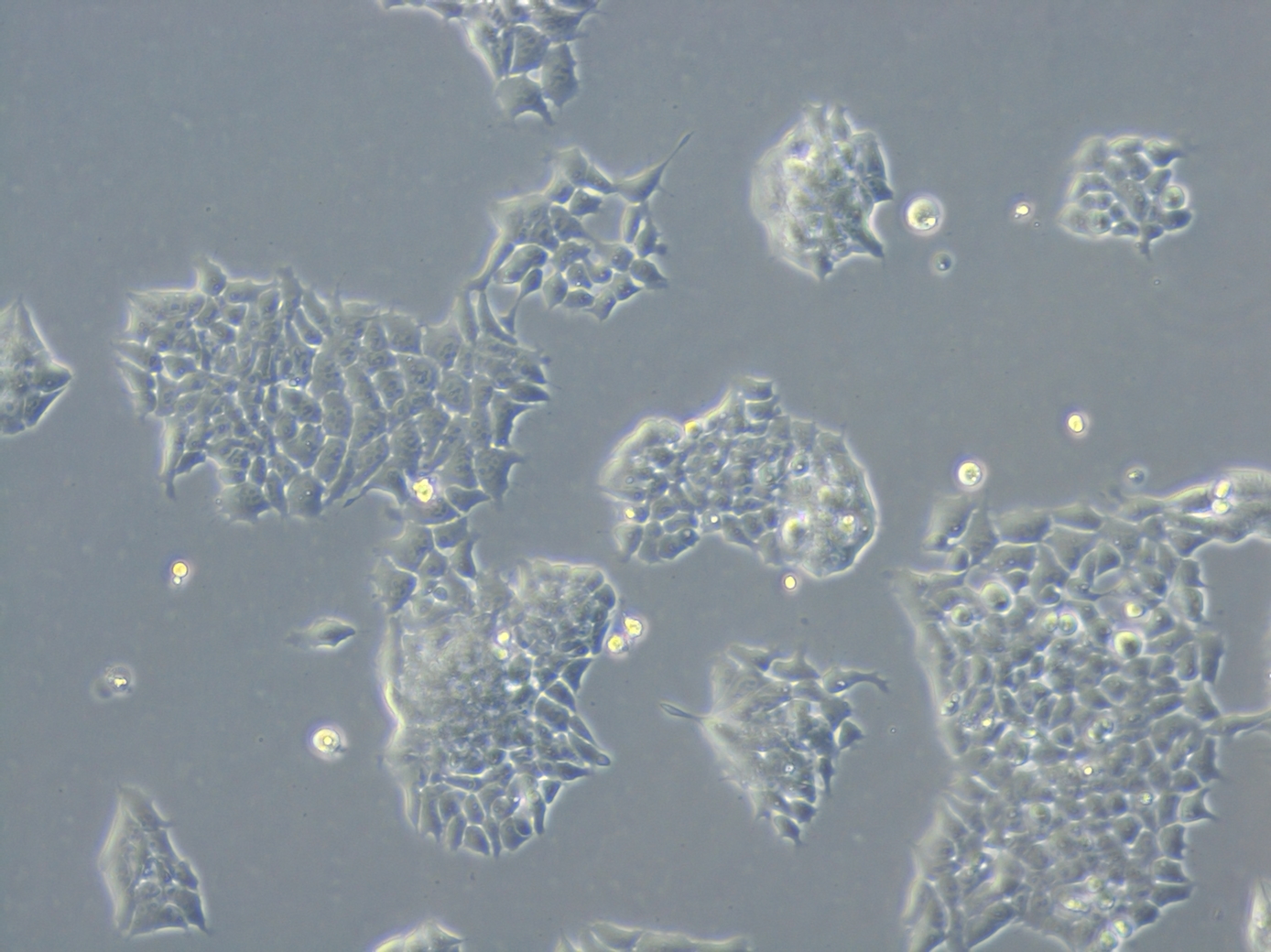

Supplement: Supplementary file 14 — Source data Fig. 3 [file 44318_2024_86_MOESM14_ESM.zip › Figure 3/Figure 3C/D6-Tead2+- 1#.jpg]

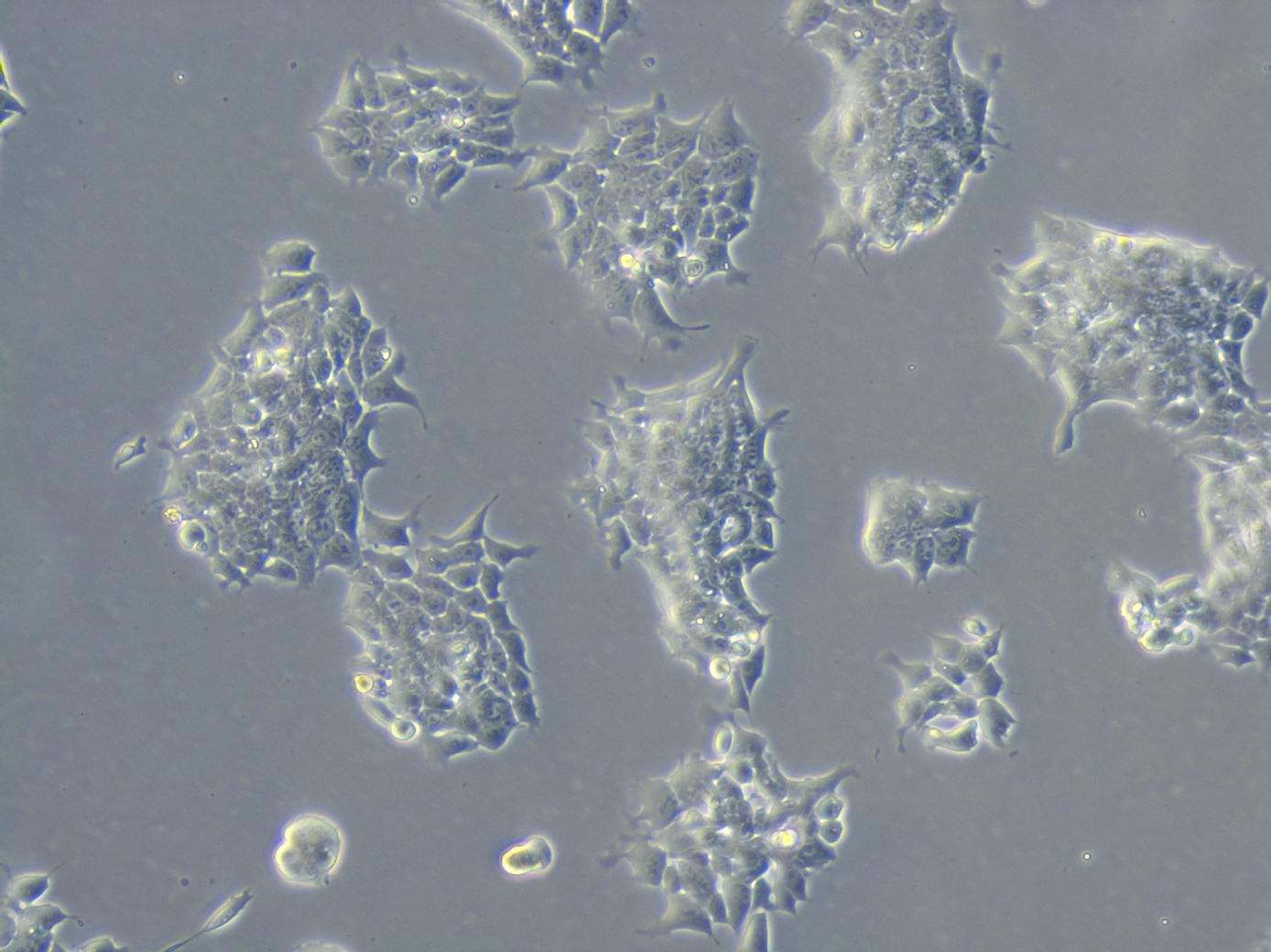

Supplement: Supplementary file 14 — Source data Fig. 3 [file 44318_2024_86_MOESM14_ESM.zip › Figure 3/Figure 3C/D6-Tead2+- 2#.jpg]

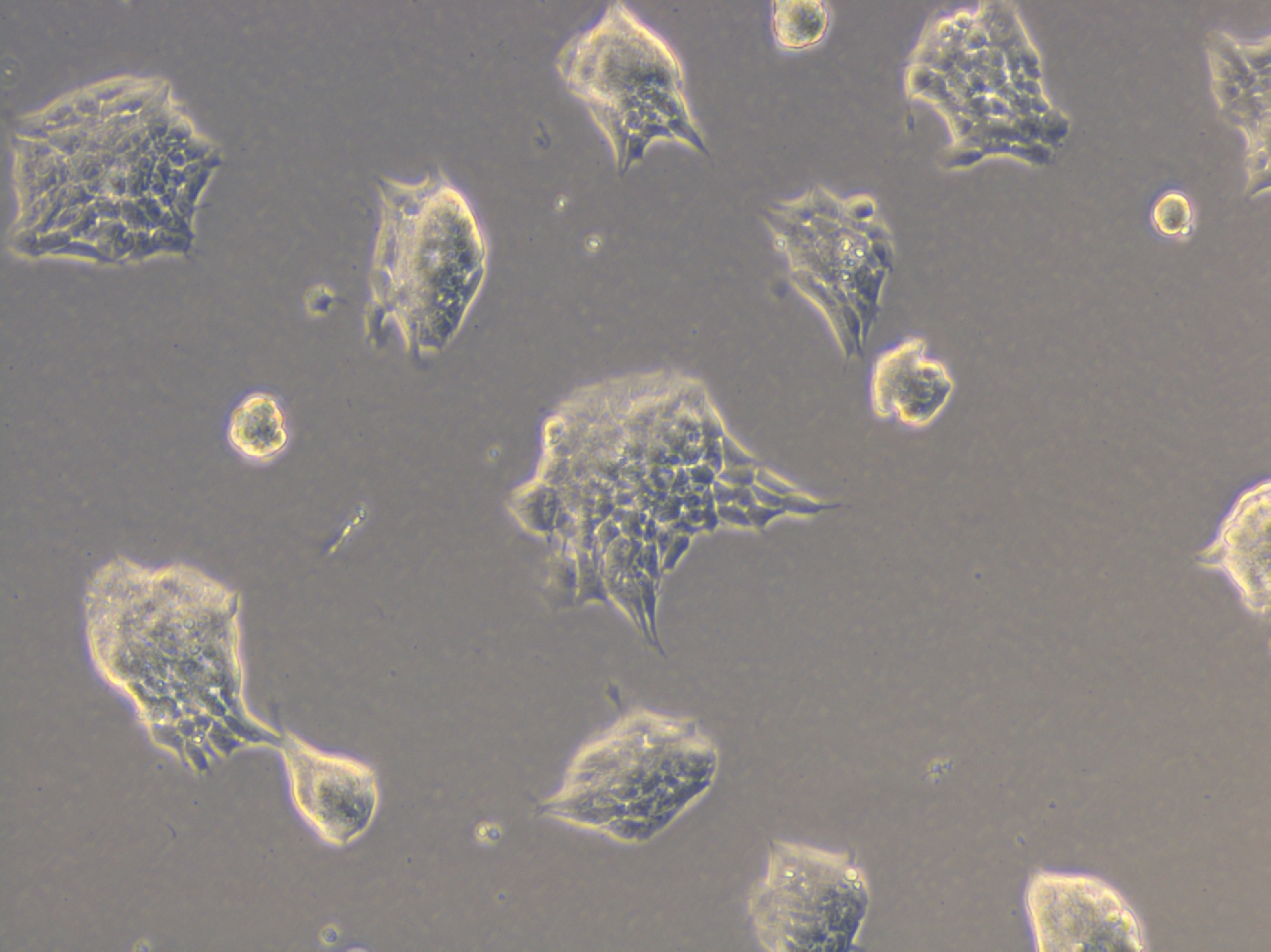

Supplement: Supplementary file 14 — Source data Fig. 3 [file 44318_2024_86_MOESM14_ESM.zip › Figure 3/Figure 3C/D6-Tead2-- 1#.jpg]

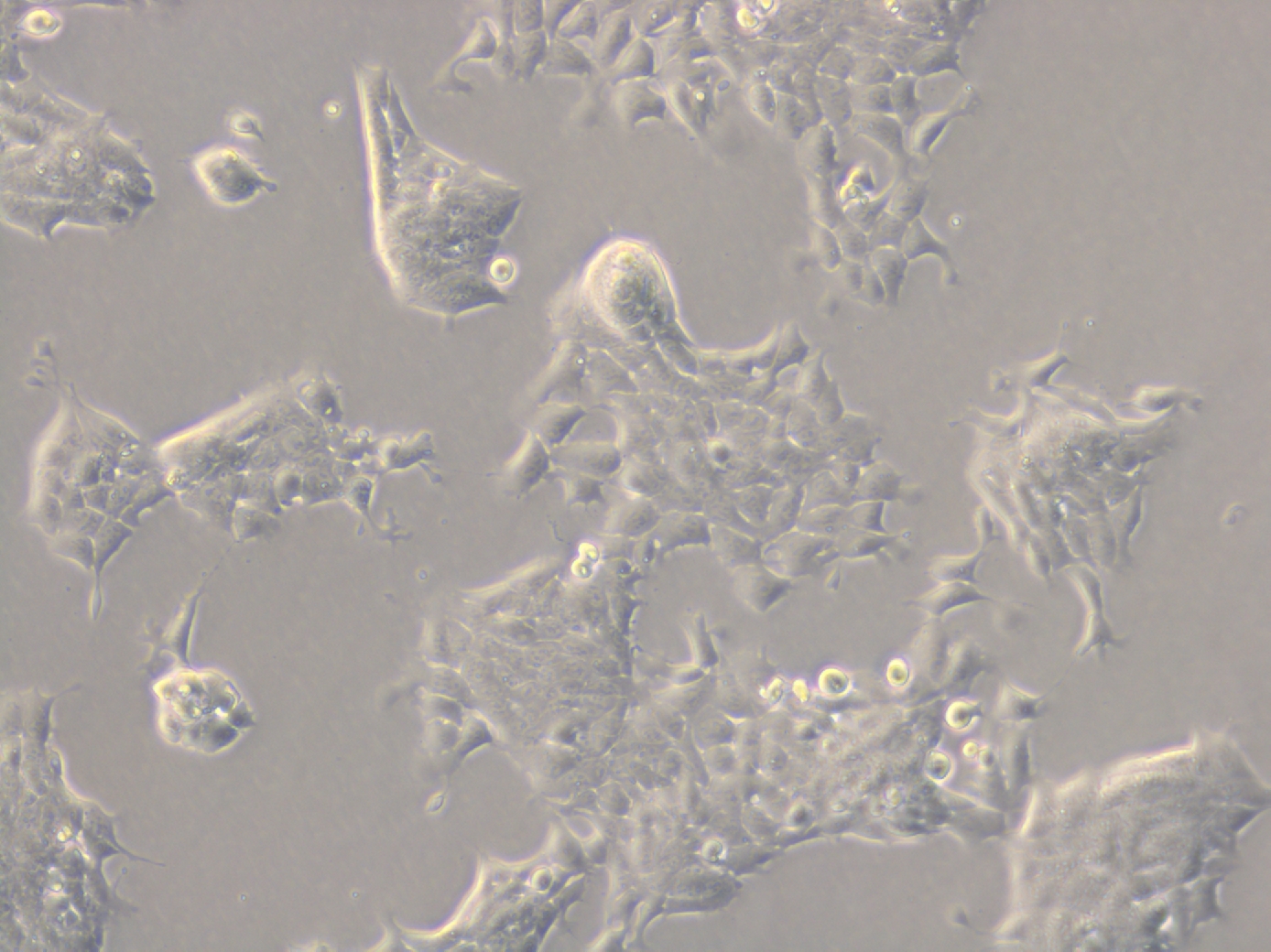

Supplement: Supplementary file 14 — Source data Fig. 3 [file 44318_2024_86_MOESM14_ESM.zip › Figure 3/Figure 3C/D6-Tead2-- 2#.jpg]

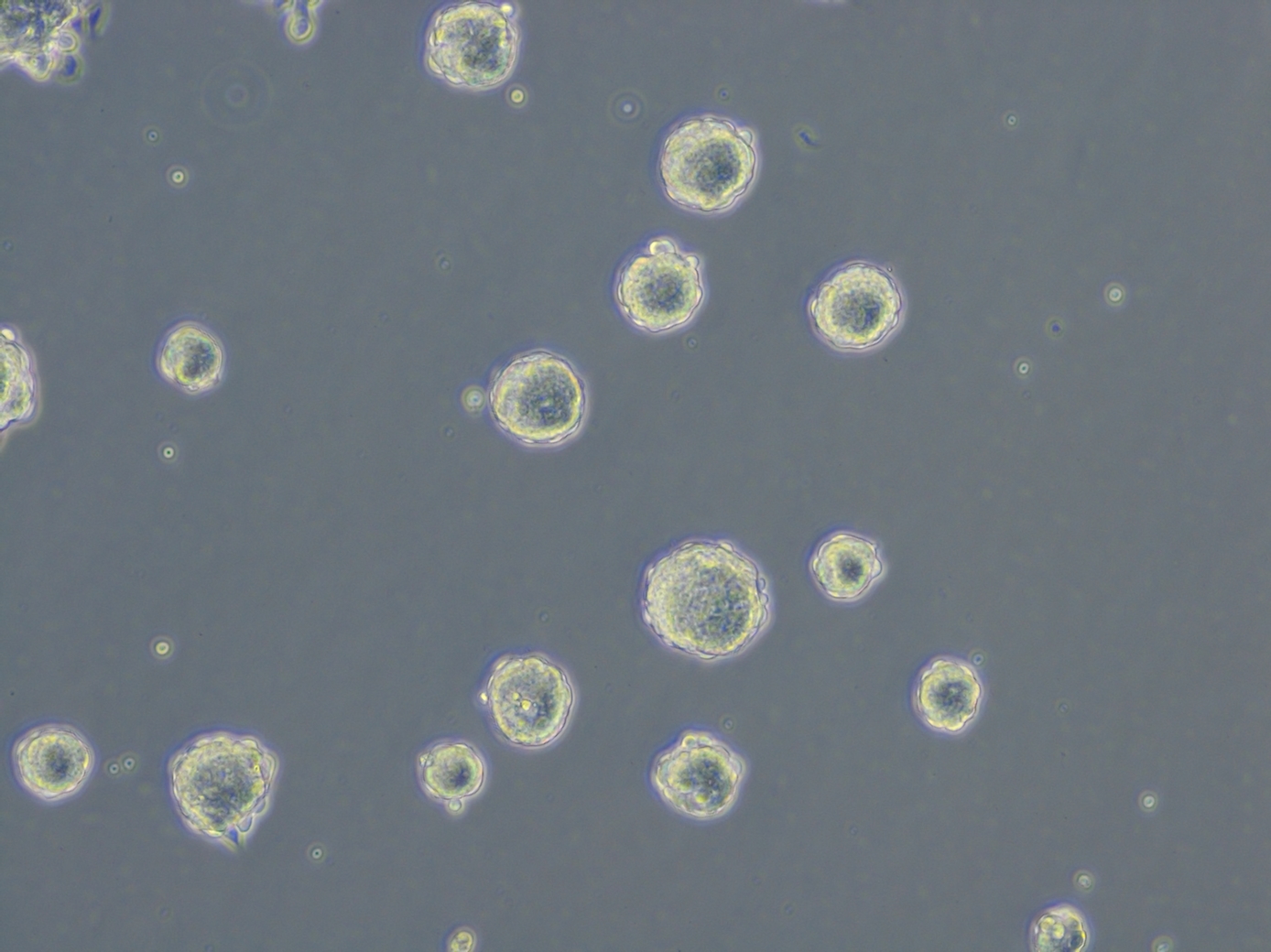

Supplement: Supplementary file 14 — Source data Fig. 3 [file 44318_2024_86_MOESM14_ESM.zip › Figure 3/Figure 3C/D6-WT.jpg]

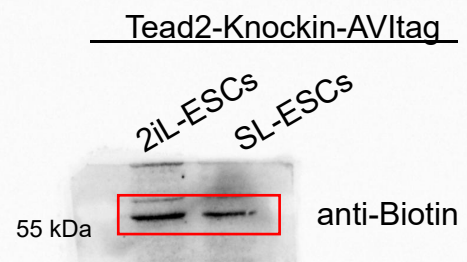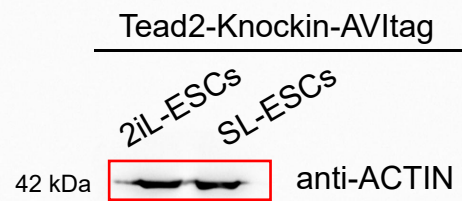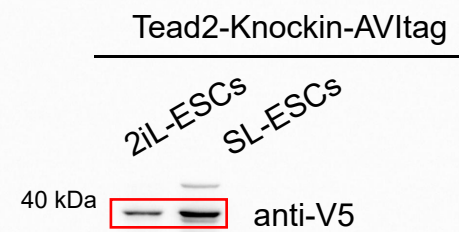

Supplement: Supplementary file 15 — Source data Fig. 4 [file 44318_2024_86_MOESM15_ESM.zip › Figure 4/Figure 4B/Western Bolt V5+ACTIN+Biotin.pdf]
