# Supplementary material for: TEAD2 initiates ground-state pluripotency by mediating chromatin looping
Source: EMBO J. 2024 Apr 11;43(10):3. doi: 10.1038/s44318-024-00086-5 (PMC11099042; doi:10.1038/s44318-024-00086-5)
Supplement: Supplementary file 18 — Expanded View Figures [file 44318_2024_86_MOESM18_ESM.pdf]

Expanded View Figures

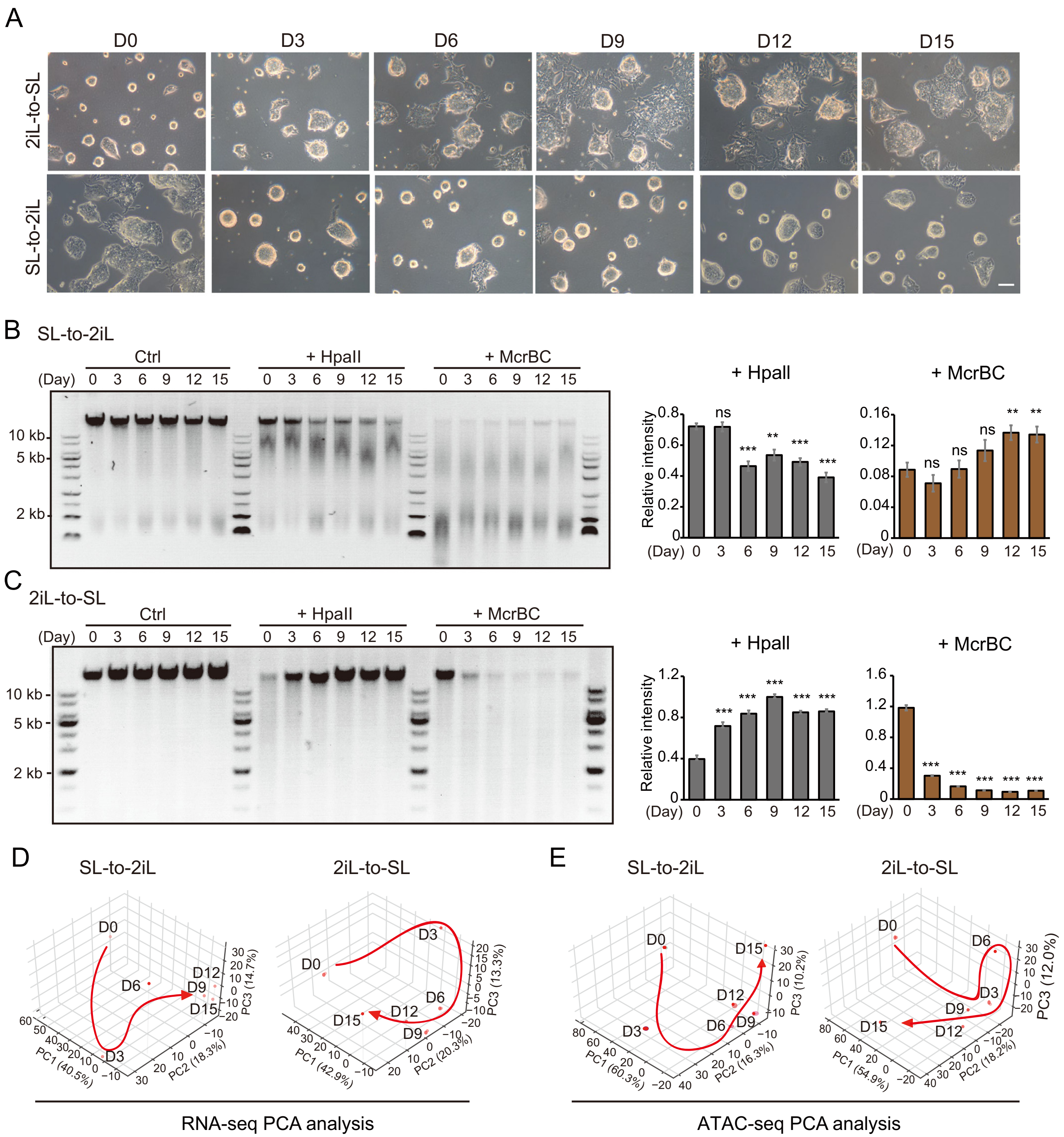

Figure EV1. Dynamic conversion between 2iL-ESCs and SL-ESCs.

(**A**) Dynamic changes of mESC morphology during the 2iL-to-SL transition from day 0 to day 15 or during the SL-to-2iL transition from day 0 to day 15. Scale bar, 100 μm. (**B**, **C**) CpG methylation levels of genomic DNA measured by digestion with the methylation-sensitive restriction enzymes *HpaII* and *McrBC* at different time points during the SL-to-2iL (**B**) and 2iL-to-SL (**C**) transitions. Quantification of DNA signals of the upper band of the agarose gel using Fiji image analysis software. Data is presented as the mean ± SD. Indicated significances are tested using Student's *t*-test analyses (**$p < 0.01$, ***$p < 0.001$). $n = 3$ replications. M, DNA ladder. (**D**, **E**) PCA of RNA-seq data (**D**) and ATAC-seq data (**E**) during the transition between 2iL-ESCs and SL-ESCs at different time points.

 © The Author(s)

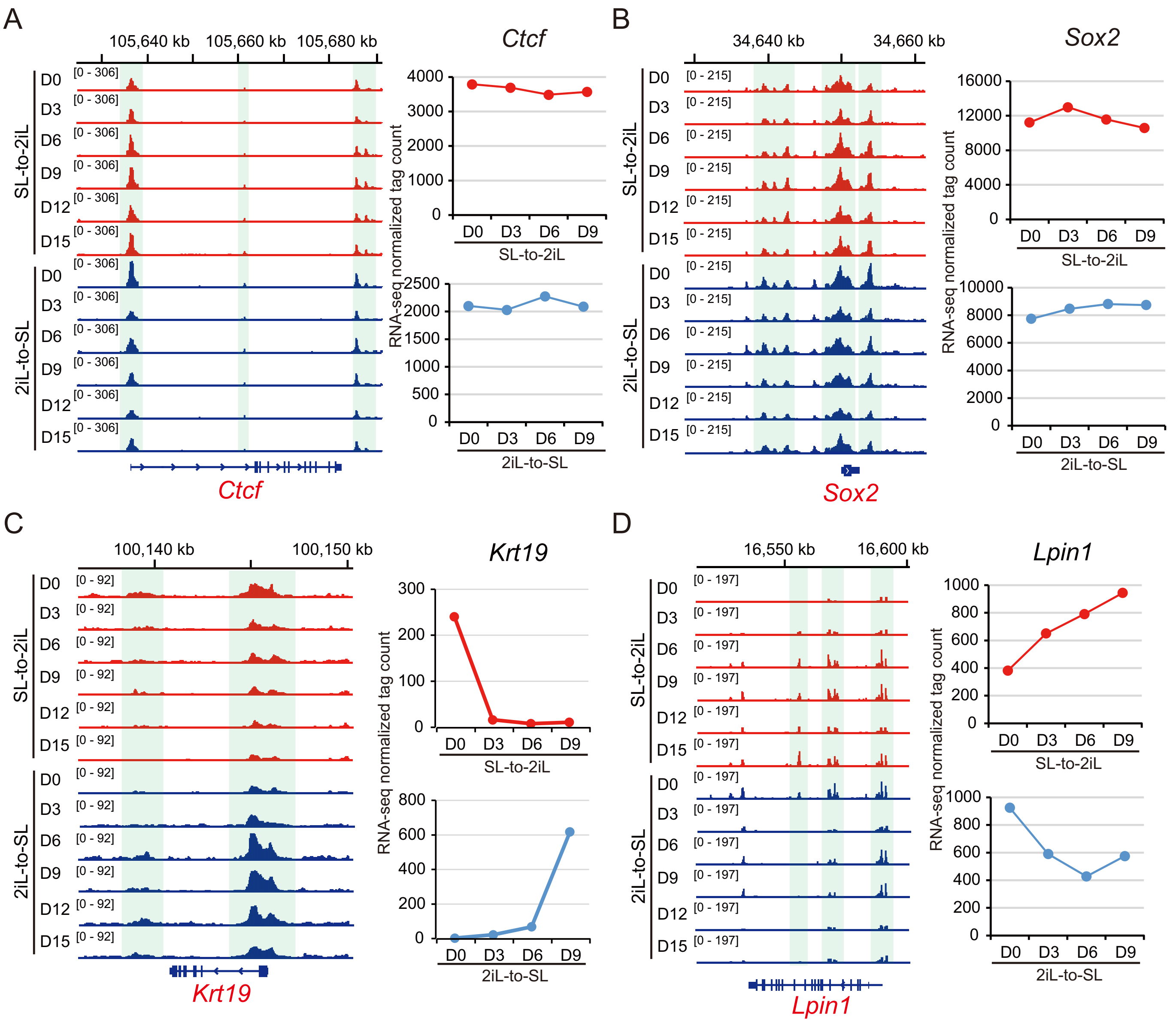

Figure EV2. Chromatin accessibility for the loci with PO, OC, and CO peaks, and expression levels of the respective genes.

(**A**, **B**) Representative loci of *Ctcf* and *Sox2* with PO peaks during the transition between SL-ESCs and 2iL-ESCs, respectively (left). Expression values of *Ctcf* and *Sox2* from RNA-seq data (right). (**C**, **D**) Representative locus of *Krt19* and *Lpin1* with Region 1/4 (**C**) and Region 2/3 (**D**) defined by ATAC-seq during the transition between SL-ESCs and 2iL-ESCs, respectively (left). Expression values of *Krt19* and *Lpin1* from RNA-seq data (right).

© The Author(s)

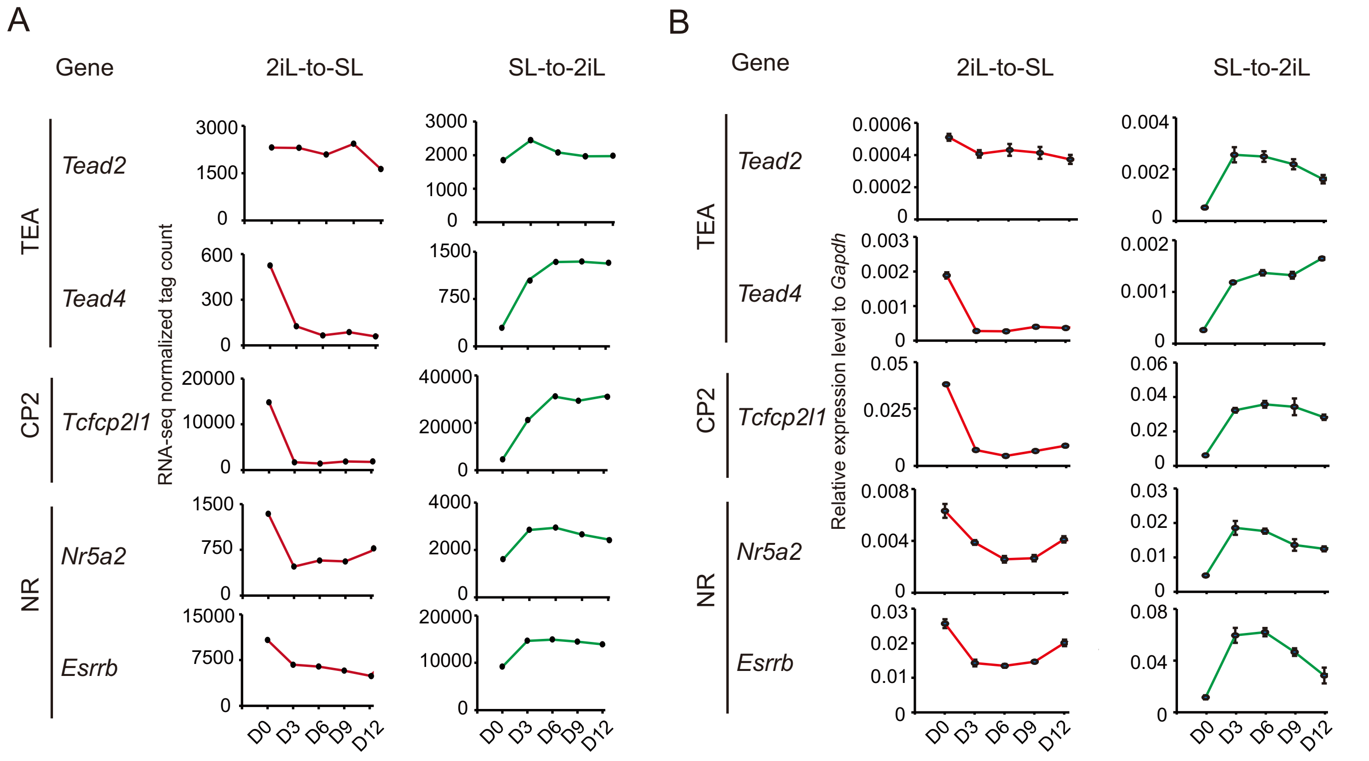

Figure EV3. Expression levels of candidate factors during the conversion between 2iL-ESCs and SL-ESCs.

(**A**) Expression values of the candidate genes from RNA-seq data. (**B**) RT-qPCR analyzing the expression levels of candidate genes from the TEA, CP2, and NR families. These are enriched in Region 2/3 during the transition between 2iL-ESCs and SL-ESCs. Data are presented as the mean ± SD. *n* = 3 biological replicates.

 © The Author(s)

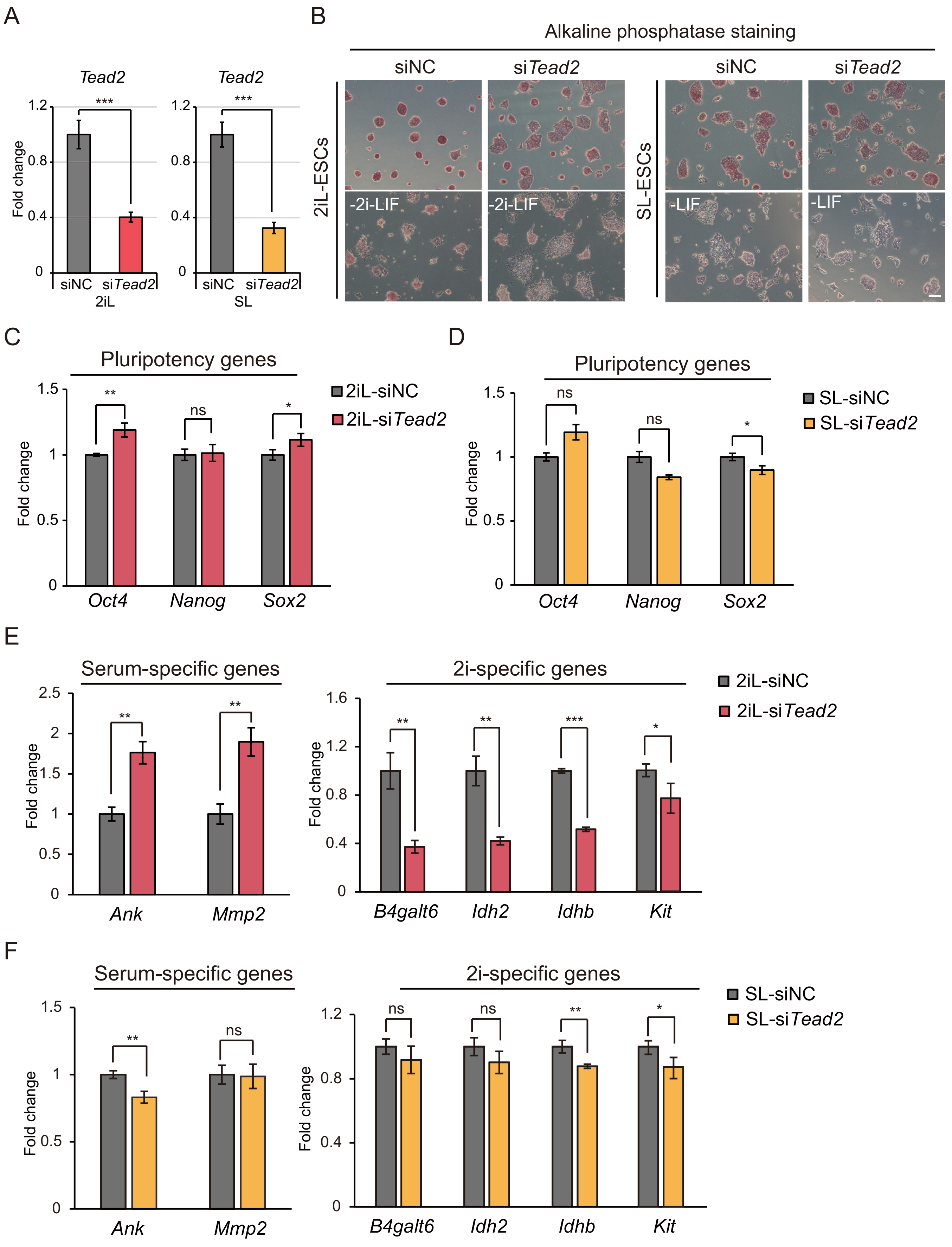
A
Tead2
Tead2
Fold change
siNC
siTead2
2iL
SL
B
Alkaline phosphatase staining
siNC
siTead2
2iL-ESCs
SL-ESCs
-2i-LIF
-LIF
C
Pluripotency genes
2iL-siNC
2iL-siTead2
Oct4
Nanog
Sox2
D
Pluripotency genes
SL-siNC
SL-siTead2
E
Serum-specific genes
2i-specific genes
Ank
Mmp2
B4galt6
Idh2
Idhb
Kit
F
Serum-specific genes
2i-specific genes

Figure EV4. Effect of TEAD2 on the ground-state pluripotency in 2iL-ESCs.

(**A**) RT-qPCR determining the *Tead2* knockdown efficiency in 2iL-ESCs and SL-ESCs. Data are presented as the mean ± SD. Indicated significances are testing using Student's *t*-test analyses (***$p < 0.001$). $n = 3$ biological replicates. (**B**) Representative images of AP staining of 2iL- and SL-ESCs and cells after 3 days of differentiation in complete medium containing 10% serum or in the absence of LIF. Cells were treated with control siRNA or siRNA targeting *Tead2*. Scale bar, 100 μm. (**C**, **D**) RT-qPCR testing the expression of pluripotent genes in 2iL-ESCs (**C**) and SL-ESCs (**D**). Data are presented as the mean ± SD. Indicated significances are tested using Student's *t*-test analyses (*$p < 0.05$, **$p < 0.01$). $n = 3$ biological replicates. (**E**, **F**) RT-qPCR testing the expression of 2i- and serum-specific genes in 2iL-ESCs (**E**) and SL-ESCs (**F**). Data are presented as the mean ± SD. Indicated significances are tested using Student's *t*-test analyses (*$p < 0.05$, **$p < 0.01$, ***$p < 0.001$). $n = 3$ biological replicates.

 © The Author(s)

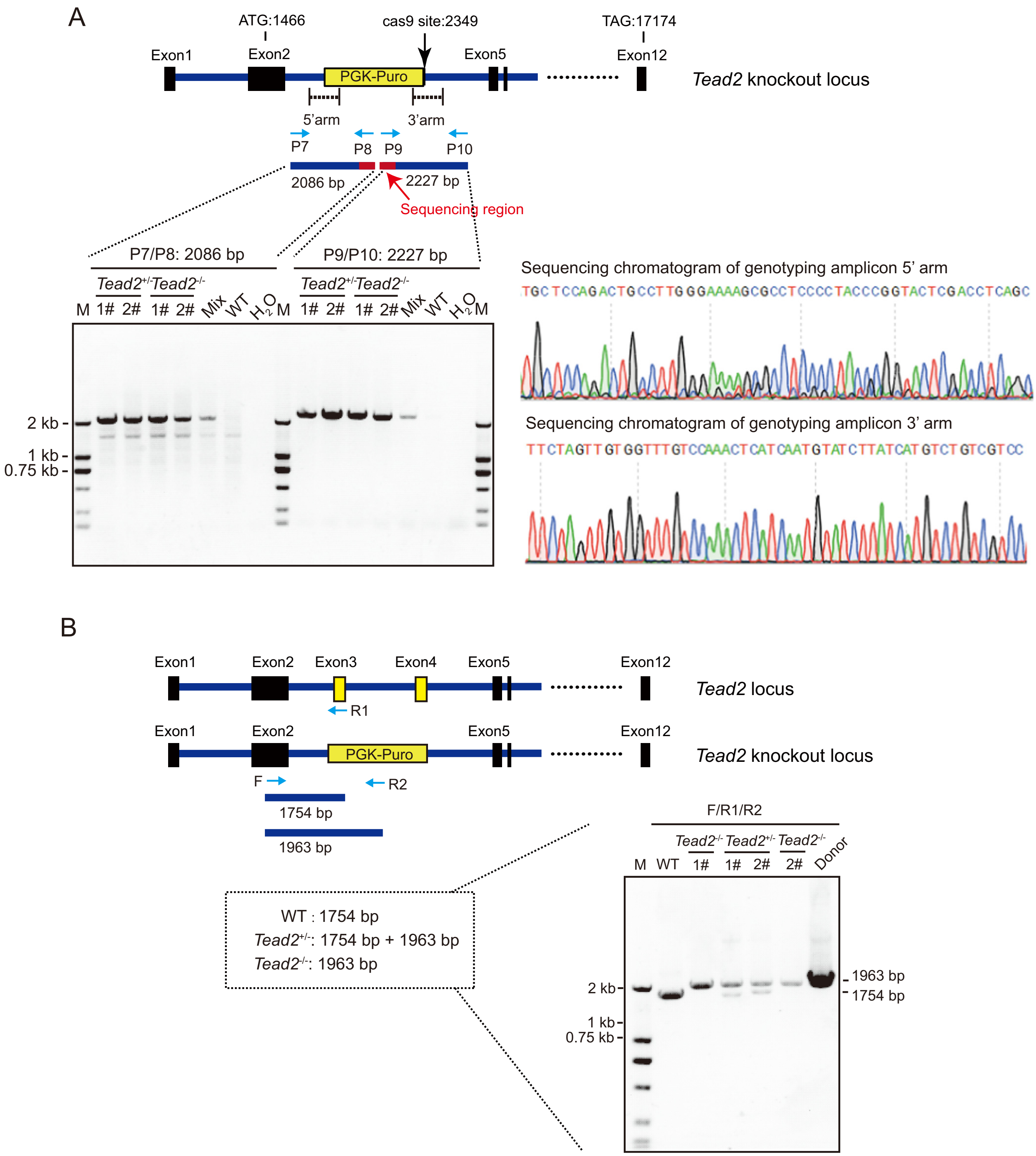

Figure EV5. PCR verification of *Tead2*-knockout in SL-ESCs.

(**A**) Genomic PCR verification of corrected clones. Primers were designed on both sides of the homologous arm to ensure that the sequence was inserted in the right position. The size of the 5′-arm terminal PCR product is 2086 bp, and the size of the 3′-arm terminal PCR product is 2227 bp. (**B**) Genomic PCR analysis identifying homozygous or heterozygous clones of *Tead2*-knockout SL-ESCs.
